# Inhibiting microglia proliferation after spinal cord injury improves recovery in mice and nonhuman primates

**DOI:** 10.7150/thno.61833

**Published:** 2021-07-31

**Authors:** Gaëtan Poulen, Emilie Aloy, Claire M. Bringuier, Nadine Mestre-Francés, Emaëlle V.F. Artus, Maïda Cardoso, Jean-Christophe Perez, Christophe Goze-Bac, Hassan Boukhaddaoui, Nicolas Lonjon, Yannick N. Gerber, Florence E. Perrin

**Affiliations:** 1 MMDN, Univ. Montpellier, EPHE, INSERM, Montpellier, France; Department of Neurosurgery, CHU, Montpellier, France.; 2 MMDN, Univ. Montpellier, EPHE, INSERM, Montpellier, France.; 3 MMDN, Univ Montpellier, EPHE, INSERM, Montpellier, France; PSL Research University, Paris, France.; 4 University of Montpellier, UMR 5221 CNRS, Montpellier, France.; 5 INSERM U1051, Institute for Neurosciences of Montpellier, Montpellier, France.; 6 Montpellier Resources Imaging (MRI), Montpellier, France.; 7 Institut Universitaire de France (IUF).

**Keywords:** spinal cord injury, microglia, proliferation, rodent, primates

## Abstract

No curative treatment is available for any deficits induced by spinal cord injury (SCI). Following injury, microglia undergo highly diverse activation processes, including proliferation, and play a critical role on functional recovery.

In a translational objective, we investigated whether a transient pharmacological reduction of microglia proliferation after injury is beneficial for functional recovery after SCI in mice and nonhuman primates.

**Methods:** The colony stimulating factor-1 receptor (CSF1R) regulates proliferation, differentiation, and survival of microglia. We orally administrated GW2580, a CSF1R inhibitor that inhibits microglia proliferation. In mice and nonhuman primates, we then analyzed treatment outcomes on locomotor function and spinal cord pathology. Finally, we used cell-specific transcriptomic analysis to uncover GW2580-induced molecular changes in microglia.

**Results:** First, transient post-injury GW2580 administration in mice improves motor function recovery, promotes tissue preservation and/or reorganization (identified by coherent anti-stokes Raman scattering microscopy), and modulates glial reactivity.

Second, post-injury GW2580-treatment in nonhuman primates reduces microglia proliferation, improves motor function recovery, and promotes tissue protection.

Finally, GW2580-treatment in mice induced down-regulation of proliferation-associated transcripts and inflammatory associated genes in microglia that may account for reduced neuroinflammation and improved functional recovery following SCI.

**Conclusion:** Thus, a transient oral GW2580 treatment post-injury may provide a promising therapeutic strategy for SCI patients and may also be extended to other central nervous system disorders displaying microglia activation.

## Introduction

Traumatic spinal cord injury (SCI) results in 0.6 to 0.9 million annual new cases worldwide 1 and induces sensory, motor, and autonomic deficits ranging from minimal dysfunctions to complete tetraplegia. There is no curative treatment available.

Following traumatism, microglia, the resident immunocompetent cells of the central nervous system (CNS) modulate neuroinflammation by releasing both detrimental and beneficial factors to their surrounding cells (for review see 2). Microglia response occurs within minutes after SCI and is followed by infiltration of neutrophils and monocyte-derived macrophages from the periphery by 6 hours and 3 days post-lesion, respectively (for review see 3, 4). Infiltrating macrophages suppress microglial activation by reducing their expression of inflammatory molecules and ability to phagocytose. Consequently, preventing chronic microglia-mediated inflammation and blocking macrophages infiltration exacerbates functional impairment after SCI 5. Notably, microglia exhibit greater SCI-induced proliferation than infiltrating macrophages 6. Moreover, microglial molecular response after SCI is characterized by an early proliferation followed by a concomitant upregulation of pro- and anti-inflammatory factors 7.

Microglia express the receptor for macrophage colony stimulating factor-1 (CSF1R). CSF1R activation regulates proliferation, differentiation, and survival of myeloid lineage cells. CSF1R inhibition triggers microglial demise *in vitro* and *in vivo* (for review see 8). Recent studies have targeted CSF1R to modulate neuroinflammatory response in physiological 9, 10 and multiple neuro-pathological conditions 11-15 (for review see 16, 17). PLX5622 is a CSF1R inhibitor that eradicates selectively and almost entirely microglia. Four weeks following traumatic brain injury (TBI) in mice, a 1-week PLX5622-treatment reduced several histopathological markers of the injury and was associated with improved motor and cognitive functions recovery 18. However, PLX5622-administration starting 3 weeks prior to TBI efficiently depleted brain microglia but failed to modify spatial learning outcomes 15. PLX5622 had also been used in mouse models of SCI 19, 20. Continuous treatment between 3 weeks prior to SCI and 35 days post-lesion worsened motor function, whilst a brief post-injury treatment between 1-6 days transiently deteriorated locomotion up to 21 days after moderate thoracic (T9/T10) contusion [50 kilodynes, kdyn] 19. PLX5622 treatment for 6 weeks post-SCI, improved cognition and depressive-like behavior recovery but did not affect the overall locomotor activity even if the regularity index and stride length were enhanced at 6 weeks following moderate/severe T10 contusion [60-70 kdyn] 20. PLX3397, another specific CSF1R inhibitor, given to mice between 7 days prior to complete T10 crush injury and 4 weeks after lesion impaired locomotor recovery, disorganized glial scar formation, increased lesion size, and reduced neuronal survival 21.

GW2580, an inhibitor of the tyrosine kinase activity of CSF1R and, to a lesser extent, other related kinases such as FMS tyrosine kinase 3 (FLT3, CD135) and oncogene KIT (c-Kit, CD117), selectively inhibits microglia/monocytes proliferation 22, 23. GW2580 treatment ameliorates neurological outcomes in animal models of multiple sclerosis 24, prion disease 25, 26, Alzheimer's disease 27, Parkinson's disease 28, lupus 29, and amyotrophic lateral sclerosis 30. Recently, we have shown that continuous GW2580-treatment between 4 weeks prior to a T9 lateral hemisection and up to 6 weeks post-lesion in mice decreases microglia proliferation, reduces gliosis, microcavity formations, and improves fine motor recovery 23. However, given that SCI is an un-predicted event, microglia manipulation pre-injury is not practical as a treatment in the clinics. To enhance translational research, it is critical to examine the effect of GW2580 treatment post-SCI not only in mice but also in nonhuman primates. Indeed, pathological responses to SCI vary considerably between rodents and primates due to differences in the neuroanatomical organization of motor and sensory systems, and neurophysiological variations 31, 32.

In the current study, we show in mice that a 1-week GW2580 oral treatment starting immediately after a T9 lateral hemisection reduces microglia proliferation, improves functional recovery, changes outcomes on myelinated fibers, and modifies glial reactivity. We then extend our investigations to *Microcebus Murinus*, a small nonhuman primate and show that orally administered GW2580 over 2 weeks after SCI transiently reduces microglia proliferation and improves motor function recovery. Finally, we used cell-specific transcriptomic analysis in mice to initiate the investigation on molecular mechanisms induced by a transient post-SCI GW2580-treatment. Notably, we identified genes that were up-regulated by SCI and further down-regulated by the treatment.

## Methods

### Animals

#### Mice

CX3CR1^+/eGFP^ transgenic mice express enhanced green fluorescent protein (eGFP) downstream of the *Cx3cr1* promoter. CX3CR1 is expressed in resident CNS microglia and circulating peripheral monocytes. Mice were maintained on a C57BL/6 background (The Jackson Laboratory, Bar Harbor, ME, USA) and housed in controlled environment (hygrometry, temperature and 12 hours light/dark cycle). Three months old heterozygote males (transcriptomics) and females CX3CR1^+/GFP^ were used.

Number of mice that underwent behavioral tests for 6 weeks: 10 controls and 10 GW2580-treated; 6 randomly selected mice of each group also underwent histological analysis. Out of these 6 mice per group, 3 in each group were also analyzed by CARS (myelin). 6 additional mice (3 in each group, 6 weeks post-injury) were dedicated to longitudinal CARS acquisition.

Five controls and 4 GW2580-treated mice were sacrificed at 2 weeks and were investigated by histology. Additionally, 6 injured animals (females, 3 months old) were dedicated to assess GW2580-treatment effects on microglia proliferation 1 week after SCI. Finally, 16 additional mice (8 controls and 8 GW2580-treated) were devoted to transcriptomic analysis.

#### Nonhuman primates

Ten adult males *Microcebus murinus* (2 years old) underwent the complete study (SCI and follow-up over 3 months post-lesion) and 6 injured animals (males, 2 years old) were dedicated to assess GW2580-treatment effects on microglia proliferation 1 week after SCI. They were all born and bred in the animal facility (CECEMA, University of Montpellier, France) and housed separately in cages (60 cm × 60 cm × 50 cm, equipped with wooden nests and enriched environment) during the entire experiment. Temperature of the animal facility was constantly kept between 24-26 °C with 55% humidity. All *Microcebus murinus* were fed 3 times a week with fresh fruits and a mixture of cereal, milk, and eggs. Water was given *ad libitum*. One day prior and after general anesthesia, animals were given mealworms to increase their protein intake.

### Spinal cord injury and post-operative cares

#### Mice

Anesthesia was induced with 3-4% isoflurane (Vetflurane®, Virbac, France) and then maintained with a mixture of 1-2% isoflurane and 1 L/min oxygen flow rate throughout the surgery. Eye gel was applied to the cornea during the surgery. A vertebral laminectomy at thoracic 9 level (T9) followed by a lateral spinal cord hemisection (HS) was done using a micro knife [10315-12, Fine Science Tools (FST)], as described previously 33. Animals were monitored over 1-hour following the surgery before returning to their cages. Bladders were emptied manually twice daily until recovery of full sphincter control. Bodyweights were monitored prior to surgery and then daily throughout the study. Animals were kept for 2- or 6-weeks post-lesion.

#### Nonhuman primates

Food and water were withdrawn 12 hours prior to surgery. Lateral HS of the spinal cord at low thoracic level (T12-L1) was done, as previously described 34. Anesthesia was induced with 3-5% isoflurane, eye gel was applied, and anesthesia was maintained with a mixture of 1-2.5 % isoflurane and 1-3 L/min O2 during the surgery. The skin was shaved and cleaned (Vetadine®, Bayer, Australia), cutaneous incision started rostrally at the costovertebral joint of floating ribs and faced 2 vertebral segments. Overlying muscles were disinserted from the midline, a laminectomy followed by a lateral spinal cord HS were done under a microscope (micro knife 10315-12, FST). Lesion quality was assessed by 2 operators and surgical area was profusely cleaned with physiological serum. Muscles and skin were sutured (vicryl, 3/0 and Filapeau 4/0, Braun, Germany, respectively). *Microcebus murinus* were placed on a temperature-controlled pad and monitored over 2 hours before returning to their cages. Animals were observed twice daily to identify potential signs of pain or distress (refusal to eat or drink, absence or decrease in grooming activity, bent posture, self-mutilation). Bladder function was controlled daily. Animals received buprenorphine (0.01mg/kg/day) and amoxicillin (10 mg/kg/jour) for 48 hours after SCI. Animals were kept for 1 week (GW2580 effects assessments) or 3 months post-lesion, bodyweights were monitored daily for 2 weeks and then weekly throughout the study.

### Treatment

#### Mice

GW2580 treatment started immediately after the injury and ended 1-week post-lesion. Mice were fed either with a standard rodent chow (A04, maintenance diet, SAFE diet, AUJY, France) or with the same diet containing 0.1% GW2580 (corresponding to 150 mg/kg per day per animal, LC Laboratories, Woburn, USA), as previously described 23.

#### Nonhuman primates

We adjusted GW2580 dose based on body surface area (BSA) of animals 35. BSA of mice and *Microcebus murinus* are approximately 0.007 and 0.016 m^2^, respectively. Thus 150 mg/kg/day (450 mg/m^2^/day) for mice corresponds to approximately 7.2 mg/day for *Microcebus murinus*. Eight animals received 7.2 mg/day of GW2580 and 8 were not treated, it includes 6 animals for GW2580 effects assessments on microglia proliferation 1 week after SCI. GW2580 treatment started immediately after SCI and ended 2 weeks post-lesion. The treatment was administered *per os*, mixed in a small quantity of applesauce; animals were monitored to ensure that they had taken the treatment.

### Bromodeoxyuridine (BrdU) experiment

We evaluated GW2580 acute effect on microglia/monocytes proliferation in both species. Cells were manually counted using the Multi-Point tool in ImageJ (National Institutes of Health, USA).

#### Mice

3 month old females underwent lateral SCI and received a daily injection of BrdU for 1 week starting immediately after lesion (i.p., 100 mg/kg, Sigma Aldrich, Gilligham, UK, in sterile saline 23), 3 animals were treated for 1 week after SCI with GW2580 and 3 were untreated. Mice were sacrificed 24 hours after the last BrdU injection and immunohistochemical analysis for BrdU and IBA1 were done 1.89 and 3.15 mm on the ipsilateral side of the lesion rostral to the injury site.

#### Nonhuman primates

We used 6 males (2 years old), all were injured and received a daily injection of BrdU for 1 week starting immediately after SCI (s.c., 100 mg/kg, Sigma Aldrich, Gilligham, UK) in sterile saline 36, 3 animals were treated for 1 week after SCI with GW2580 and 3 were untreated. Lemurs were sacrificed 24 hours after the last BrdU injection and immunohistochemical analysis for BrdU and IBA1 were done 5 and 5.28mm rostral to the lesion.

### Behavioral assessments

#### Mice

Assessments were done at 2 weeks, 1 week and 1 day prior to injury followed by 3 days, 5 days and then once a week up to 6 weeks after lesion (n = 10 for untreated and GW2580 groups). Dynamic walking pattern (CatWalk™, XT Noldus, Wageningen, The Netherlands) analysis were done, as earlier reported 23, 33, 37. Six CatWalk™ runs per animal were analyzed per time point. CatWalk™ data analyses were done using CatMerge (Innovationet, Tiranges, France).

#### Nonhuman primates

1-month prior to surgery, habituation to manipulation by the operators and behavioral tests were done 3 times per week to acquire accurate pre-operative values. After injury, tests were done at 1, 3, 5, 7, 10- and 14-days post-surgery and then once a week until 3 months after lesion. Three tests to evaluate the gait and motor activity were done. CatWalk™ Noldus, Wageningen, The Netherlands): 6 runs per animal with at least 3 un-interrupted step cycles were acquired, as previously described 34. For each animal, values obtained following lesion were normalized to those obtained prior to surgery (results are expressed as percentage of the median pre-operative value). To better assess fine motor recovery, we designed a ladder made of wooden bars with 4 different diameters (15, 10, 5 and 3 mm, bottom to top, **Figure 5F**). Animals were placed on the bottom of the ladder and video recorded while climbing toward their nest located at the top of the ladder. Two parameters were quantified for each paw to grade movements (overall movement of a given hind limb and capability to grip) on a scale of 5 and then summed to obtain a value reaching a maximum of 10 in case of normal movements (**Table 1**). To better assess balance and grip recovery, we used a metal bar (diameter: 3 mm) located at 20 cm from the ground within an empty Plexiglas test arena (**Figure 5H**). Primates were placed on the bar, and experimenters gently rotated the bar to evaluate the capacity of the animal to use its hind paw and to grip the bar. Animals were video recorded during the whole procedure. Each animal was scored through a motor grading of each hind paw. Final score resulted from the sum of the scores of the overall capacity for the hind paw ipsilateral to the lesion to move toward the bar (0 = no movement, 1 = incomplete movement, 2 = complete movement) and to grip the bar (0 = no grip, 1 = incomplete grip, 2 = complete grip). Video recordings were acquired with a high-resolution camera (HD 1080P, Logitech, Newark, CA, USA) and blindly analyzed by two independent experimenters.

### *Ex vivo* diffusion-weighted magnetic resonance imaging (DW-MRI)

At 3 months post-injury for nonhuman primates, animals were injected with a lethal dose of ketamine (150 mg/kg, Merial, Lyon, France). Animals were then perfused intracardially with cold phosphate saline buffer (PBS, 0.1M, pH 7.2) followed by cold 4% paraformaldehyde (PFA, pH 7.2, Sigma Aldrich, Darmstadt, Germany) in 0.1 M PBS. Spinal cords were then dissected and further post-fixed in the same fixative for 2 additional hours and then stored in 1% PFA until *ex vivo* MRI acquisition.

For *ex vivo* acquisition, spinal cords were placed in Fluorinert FC-770 liquid (3M™ Electronic Liquids, Saint Paul, USA) in a 4-mm-diameter glass tube surrounded by a custom-made solenoid coil dedicated to SCI investigations 37, 38. The coil was placed in the 9.4 Tesla apparatus (Agilent Varian 9.4/160/ASR, Santa Clara, California, USA) associated with a VnmrJ Imaging acquisition system (Agilent, USA). Axial *ex vivo* MRI scans were done using Single Echo Multi Slices (SEMS) sequence (TR = 1580 ms; TE = 30.55ms; AVG = 30; FOV = 10 mm*10 mm; 36 slices; thickness = 1 mm; gap = 0 mm; acquisition matrix (NREAD*NPHASE) = 128*128). Diffusion gradients were applied in 3 directions including the rostro-caudal axis and 2 directions perpendicular to the spinal cord (Gs = 10 G/cm; delta = 6.844 ms; separation = 15.05 ms; b-value = 499.21 s/mm²). The same images were also acquired without applying diffusion gradient (Gs = 0 G/m^-1^). MRI visualizations and segmentations were done manually using Myrian Software (Intrasense, Montpellier, France), as described previously 34. Longitudinal (LADC) and transversal apparent diffusion coefficients (TADC) were measured on a 2 cm segment centered on the lesion site. Immediately after MRI acquisitions, spinal cords were rinsed in 0.1 M PBS, cryoprotected in 30% sucrose, embedded in Tissue Tek (Sakura, Alphen aan den Rijn, The Netherlands), frozen and kept at -20 °C.

### Histology

Mice were injected with a lethal dose of tribromoethanol (500 mg/kg, Sigma-Aldrich Darmstadt, Germany) and perfused intracardially and their spinal cord processed and frozen as described above for primates. Serial 14-µm-thick axial spinal cord cryosections (Microm HM550, Thermofisher Scientific, Waltham, USA) were collected on Superfrost Plus© slides. For nonhuman primates all sections were collected conversely to mice where 1 section each 3 sections was collected.

#### Luxol fast blue and neutral red staining in nonhuman primates

Luxol fast blue staining was done as previously described 39. Briefly, sections were placed 5 min in 95% ethanol and then incubated in 0.1% Luxol fast blue under mild shaking [12 hours, room temperature (RT)]. Slides were rinsed in milli-Q water (1 min), then placed in lithium carbonate (1 min, 0.05%) and finally washed in tap water (1 min). Subsequently, slides were incubated for 10 min in 0.5% neutral red solution, 5 min in 100% ethanol and washed twice for 10 min in xylene. All slides were coverslipped using Eukitt (Sigma Aldrich, Darmstadt, Germany). Quantifications of lesion extension and volume were done on a 1-cm segment centered on the lesion site; sections were analyzed at 210 µm intervals in nonhuman primates. The lesion area was expressed as a percentage of the total surface area; spared white and grey matters were measured (mm^2^).

### Fluoromyelin staining

14 µm-thick axial *Microcebus murinus* spinal cord cryosections were incubated 20 min with fluoromyelin (1:200, Invitrogen, Carlsbad, USA), then rinsed 3×10 min in PBS and mounted with fluorosave (Dako, Glostrup, Denmark). One pictures of 600 µm × 400 µm was acquired in both lateral *funiculi* (ipsilateral and contralateral to the lesion). In each picture 3 fields of 40 µm × 40 µm were quantified. ImageJ software was used (National Institutes of Health, USA) for quantifications. Quantifications were done 1.68 mm caudal to the lesion epicenter.

#### Immunohistochemistry in mice and nonhuman primates

Transversal 14-µm-thick axial spinal cord sections were washed twice in 0.1 M PBS, treated for 10 min in 0.1M PBS containing 20 mM lysine (pH 7.2) and 15 min in hydrogen peroxide (1% in 0.1 M PBS, Sigma Aldrich, Gilligham, UK). Sections were washed twice in 0.1 M PBS and blocked for 2 hours with 0.1M PBS containing 1% bovine serum albumin and Triton X-100 (0.1%) (both from Sigma Aldrich, Gilligham, UK) and then incubated for 48 hours at 4 °C with the primary antibody, excepted for negative controls. Sections were rinsed with 0.1M PBS (30 min) and incubated in 1:500 dilution of the corresponding biotinylated secondary antibody (2 hours, RT). Sections were rinsed with 0.1 M PBS (30 min). For amplification, Avidin Biotin Complex solution (Vector Laboratories Ltd. Peterborough, UK) diluted at 1:100 in 0.1 M PBS was added on slides and incubated (1 hour, RT). Then, sections were rinsed in 0.1 M Tris (pH 7.6, RT). Protein expression was visualized using DAB peroxidase substrate kit (Vector Labs, Burlingame, USA). The reaction was stopped by rinsing the sections in 0.1M Tris (3x10 min). Slides were dehydrated in increasing concentrations of ethanol and then xylene. Coverslips were applied using Eukitt (Sigma Aldrich, Darmstadt, Germany). For BrdU detection, sections were first pre-incubated in 2N HCl (hydrogen chloride, 30 min) for DNA (deoxyribonucleic acid) denaturation followed by 0.1 M pH 8.5 sodium borate buffer washes (Sigma Aldrich, Gilligham, UK) (3x10 min). Sections were then incubated with a combination of rabbit anti-IBA1 and rat anti-BrdU antibodies and then rinsed with PBS (3×10 min). Sections were then incubated in a solution containing the corresponding anti-rat fluorescent and anti-rabbit biotinylated secondary antibodies. Second, a streptavidin fluorescent conjugated antibody was used to amplify IBA1 immunodetection. Sections were cover slipped using fluorescence mounting medium (Dako, Glostrup, Denmark).

#### Antibodies

Primary antibodies: anti-ionized calcium-binding adapter molecule 1 (IBA1, peroxidase staining, 1:1000 for mice, 1:200 for *Microcebus*, Wako Pure Chemical Industries, Japan) and anti-BrdU antibody (1:500; Abcam, Cambridge, UK).

Secondary antibodies: Biotinylated anti-rabbit (1:500, Invitrogen, Carlsbad, USA). Fluorescent anti-rat (Alexa 594) and biotinylated anti-rabbit coupled with streptavidin fluorescent conjugated antibody (Alexa 488) (1:1000, both Life Technologies, Carlsbad, USA).

### Microscopy and quantifications

#### Brightfield microscopy

We used NanoZoomer RS slide scanner with constant light intensity and exposure time and NanoZoomer Digital Pathology System view software (Hamamatsu, Hamamatsu, Japan). To quantify SCI and GW2580 treatment-induced changes in IBA1 expression, the mean optical density (OD) was measured along the spinal cord, as previously described (ImageJ, National Institutes of Health, USA) 23, 33, 34, 37. To minimize bias in staining intensity, all immunostainings for a given antibody and a given time-point were done in parallel. For all antibodies used, expression levels were analyzed in at least 40 and 16 axial sections throughout the lesion segment of the spinal cord at 210 µm and 630 µm intervals for *Microcebus Murinus* and mice respectively. OD quantifications included grey and white matters (excluding the dorsal *funiculus*) and dorsal *funiculus*. Background was subtracted from OD values of each section. All quantifications were done blindly.

#### Fluorescent microscopies

Images were obtained using the Axio Imager 1 microscope (Zeiss, Oberkochen, Germany). Settings were kept constant for all acquisitions. BrdU and IBA1(or eGFP^+^): BrdU/IBA1 double positive cells were manually counted in 2 sections per animal located 1.89 & 3.15 mm (mice) and 5 & 5.28 mm (lemur) on the ipsilateral side of the lesion rostral to the epicenter using ImageJ software (National Institutes of Health, USA). Fluoromyelin pictures were acquired with THUNDER Imager 3D (Leica, Wetzlar, Germany; lens ×63).

#### Coherent anti-stokes Raman scattering (CARS)

We used LSM 7 MP optical parametric oscillator (OPO) multiphoton microscope (Zeiss, Oberkochen, Germany) with an upright Axio Examiner Z.1 optical microscope associated with a femtosecond Ti: sapphire laser (680-1080 nm, 80 MHz, 140 fs, Chameleon Ultra II, Coherent, France) pumping a tunable OPOs (1000-1500 nm, 80 MHz, 200 fs, Chameleon Compact OPO, Coherent, France) to acquire CARS images. We imaged axial spinal cord sections (14µm) and longitudinal sections (22 µm) at 6 weeks after injury in mice. Axial sections (14 µm thickness) were taken at 3 months after injury in primates. A ×20 water immersion lens (W Plan Apochromat DIC VIS-IR) with the following characteristics was used: 1024×1024 pixels frame size, scan speed of 6 (zoom ×1.2) and 8 (mosaic, zoom x3, PixelDwell 3.15 and 1.27 μs/scan, respectively) and either a zoom ×1.2 or ×3. CARS excites the CH2 vibrational mode at 2845 cm^-1^ and CH2 bonds are found in lipids 40. Excitation wavelengths were 836 and 1097 nm (synchronized Ti-saphire and OPO, respectively) and the signal was detected at 675 nm (filter from 660-685 nm). Acquired images were a stack of 3 µm (3 slices). Myelin degradation and myelin sheaths density were scored and manually quantified.

### Neuromuscular junction labelling

Following animal's perfusion, *gastrocnemius-soleus-plantaris* muscular complex were collected for neuromuscular junction labelling using the method described by Karnovsky and Roots 41. Axial sections (16 µm) of the entire muscle complex were analyzed. Every 15 sections in the *gastrocnemius*, muscle fibers were manually segmented by a blinded experimenter, their surface were quantified and NMJs number was counted.

### Statistics

Statistical tests were done using GraphPad Prism (GraphPad software 5.03, USA). Significance was accepted at p ≤ 0.05. Results are expressed as mean ± standard error of the mean (SEM). For behavioral analysis, repeated measures 2-Way ANOVA with Bonferroni post-hoc tests were used. For all other analysis student's unpaired t-test was used.

### Transcriptomic analyses

We used Fluorescence Activated Cell Sorting (FACS) to isolate eGFP+ microglia from a 1cm-spinal cord segment centered on the lesion site, as previously described 7. Briefly, treated and untreated spinal cord injured CX3CR1^+/GFP^ mice were anesthetized (tribromoethanol, 500 mg/kg) and intracardially perfused with 0.1 M RNAse-free phosphate base saline (PBS, Invitrogen, Carlsbad, USA). Spinal cords were dissociated in an enzymatic cocktail [750 µl PBS, 100 µl of 13 mg/ml trypsin, 100 µl of 7 mg/ml hyaluronidase, 50 µl of 4 mg/ml kinurenic acid (all from Sigma Aldrich, Saint Louis, USA), and 20 µl of 10 mg/ml DNAse I (Roche, Rotkreuz, Switzerland)] for 30 min at 37 °C. Cell suspension was separated (40 µm sieve, BD Biosciences, Franklin Lakes, USA), re-suspended in 0.9 M sucrose and centrifuged (20 min, 750 g). Pellet was re-suspended in 500 µl of 7AAD 1 µl/ml (Sigma Aldrich, Saint Louis, USA) and eGFP^high^ expressing cells, that we have previously shown to correspond to microglia 7, were sorted using FACS ARIA (BD Biosciences, Franklin Lakes, USA), equipped with a 488nm Laser Sapphire 488-20. Size threshold was used to eliminate cellular debris (**Supplementary Figure 3**). Sorted microglia were centrifuged (5 min, 700 g) and re-suspended in 250 µl of RLT lysis buffer (Qiagen, Maryland, USA) and 1% beta-mercaptoethanol. RNA was isolated using the RNeasy Mini Kit, (Qiagen, Maryland, USA, with DNAse) and its quality assessed (Agilent 2100 bioanalyzer, RNA 6000 Pico LabChip, Palo Alto, USA). Only RNA with a RNA integrity number (RIN)>7 were further processed. RNA-Seq was performed on 3 biological replicates per condition (each replicate for both untreated and treated conditions consisted on pooled 1-cm spinal cord segments from at least 2 animals representing a minimum of 16.000 microglia). For reverse transcription and cDNA amplification, we used the SMART-Seq v4 kit (Takara Bio USA, Mountain View, CA, USA) according to manufacturer's specifications, starting with 2ng of total RNA as input. 200 pg of cDNA were used for library preparation using the Nextera XT kit (Illumina, San Diego, CA, USA). Library molarity and quality was assessed with the Qubit and Tapestation using a DNA High sensitivity chip (Agilent Technologies, Santa Clara, CA, USA). Libraries were pooled and loaded for clustering on 1 lane of a Single-read Illumina Flow cell (for an average of 50 million of reads per library) (Illumina, San Diego, CA, USA). Reads of 100 bases were generated using the TruSeq SBS chemistry on an Illumina HiSeq 4000 sequencer (Illumina, San Diego, CA, USA). FastQC was used to assess sequencing quality control, the reads were mapped with the STAR aligner and biological quality control and summarization were done with PicardTools. The counts were produced from aligned reads by the Python software htseq-count with the reference .gtf file. https://htseq.readthedocs.io/en/release_0.11.1/count.html. Normalization and differential expression analysis were performed with the R package edgeR, for the genes annotated in the reference genome http://www.ncbi.nlm.nih.gov/pmc/articles/PMC2796818/. Very lowly expressed genes were filter out to keep only genes that were sufficiently expressed (above 10 in the 3 biological replicates); filtered data were normalized according to the RNA composition and library size. Differentially expressed genes were estimated using a GLM approach (General Linear Model), negative binomial distribution and a quasi-likelihood F-test. Differentially expressed transcripts were defined with a criterion of a 2-fold and greater difference plus a significant p-value with false discovery rate (FDR) ≤0.05. Pathway analysis was done using MetaCore, Clarivate Analytic, Philadelphia, USA.

### Ethic committee approval

Experiments were approved by the Veterinary Services Department of Hérault, the regional ethic committee n°36 for animal experimentation, and the French Ministry of National Education, Higher Education and Research (authorizations; mice: n°34118 and nonhuman primates n° APAFIS#16177-2018071810113615v3). Experimental procedures followed the European legislative, administrative and statutory measures for animal experimentation (EU/Directive/2010/63 of the European Parliament and Council) and the ARRIVE guidelines.

## Results

### Transient post-injury CSF1R blockade in mice enhances motor recovery after SCI

As a pre requisite we first examined whether a transient (1 week) oral GW2580-treatment administered after a T9 lateral spinal cord hemisection reduces microglia proliferation as we previously demonstrated using the same dosage but with a treatment starting before the lesion 23. GW2580 was orally administered for 1 week to CX3CR1^+/GFP^ females following a lateral hemisection of the spinal cord. Concomitantly, BrdU injections were daily performed. At the end of the treatment *i.e*. 1 week after SCI, microglia (eGFP^+^ cells), proliferating cells (BrdU^+^) and proliferating microglia (eGFP^+^/BrdU^+^ cells) were quantified in untreated and GW2580-treated animals. For each animal, quantifications were done on 2 sections located 1.89 and 3.15mm on the ipsilateral side rostral to the lesion epicenter. GW2580-treated and untreated groups presented a similar number of proliferating cells, indeed densities of 143±30 and 203±22 of BrdU^+^ cells per mm^2^ were counted, respectively. Likewise, no difference in microglia densities was observed since 520±49 and 615±18 eGFP^+^ cells per mm^2^ were counted in the GW2580-treated and untreated groups respectively. Conversely, the density of proliferative microglia (eGFP^+^/BrdU^+^ cells) in the GW2580-treated group was reduced as compared to the non-treated treated group since 59±11 and 103±8 cells/mm^2^ (*, p = 0.012) were counted, respectively. Thus, 1-week GW2580-treatment after SCI reduces microglia proliferation SCI in mice.

**Figure 1:**
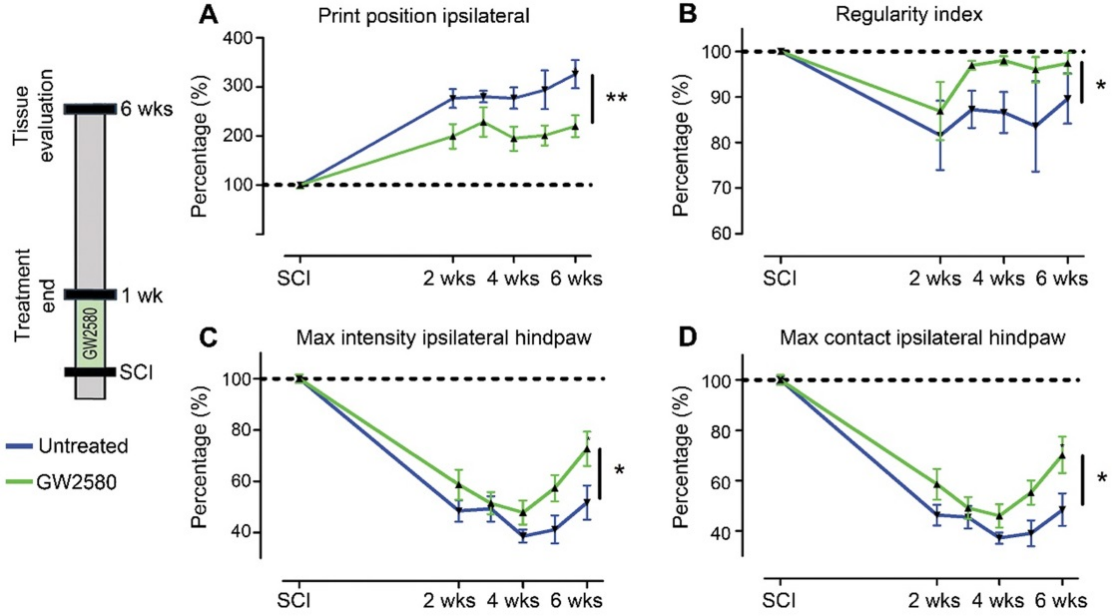
** Transient CSF1R blockade after SCI in mice improves motor function recovery.** CatWalk^TM^ behavioral analysis (**A-D**). Values were normalized to those obtained by the same animal prior to the lesion (represented as dash lines, 100%). Graphs display in both GW2580-treated and untreated groups the print position of the paw ipsilateral to the lesion (p = 0.007, f = 9.38 and Df = 1) (**A**), the regularity index (p = 0.032, f = 5.37 and Df = 1) (**B**), the max intensity of the ipsilateral hind paw (p = 0.015, f = 7.20 and Df = 1) (**C**), and the maximum intensity at max contact of the ipsilateral hind paw (p = 0.012, f = 7.73 and Df = 1) (**D**). In all graphs, results obtained by untreated mice are in blue and GW2580-treated mice in green. Data are mean ± SEM per group. wks = weeks. Repeated measures two-Way ANOVA followed by Bonferroni post-hoc tests, *p < 0.05 and **p < 0.01. p = pvalue; f = f-values and Df = degree of freedom. Number of mice: n = 10 in each group.

We then studied the effect of reducing microglia proliferation after SCI on functional recovery. We examined motor recovery over 6 weeks post-injury. GW2580 treatment improved both static and dynamic parameters. The “print position” ipsilateral to the lesion recovered better in the GW2580 group (**Figure 1A**). The “print position” reflects the distance between the front and the hind paws located on the same side in a step cycle and thus measures normality of the hind paw motricity. The “regularity index” measuring inter-paw dynamic coordination also recovered better in the GW2580 group (**Figure 1B**). Indeed, almost full coordination from 3 weeks after SCI was observed in the treated group inversely to the untreated group. The “max intensity” (print maximum intensity) of the hind paw ipsilateral to the lesion (**Figure 1C**) and the “max contact” (max intensity of the paw at max contact) (**Figure 1D**) also recovered better in GW2580-treated mice than in the untreated controls. Improvements appeared at 4 weeks post-lesion. Therefore, a transient 1-week GW2580 treatment immediately after SCI promotes motor function recovery in mice.

### GW2580 treatment after SCI in mice modulates microglial reactivity

**Figure 2:**
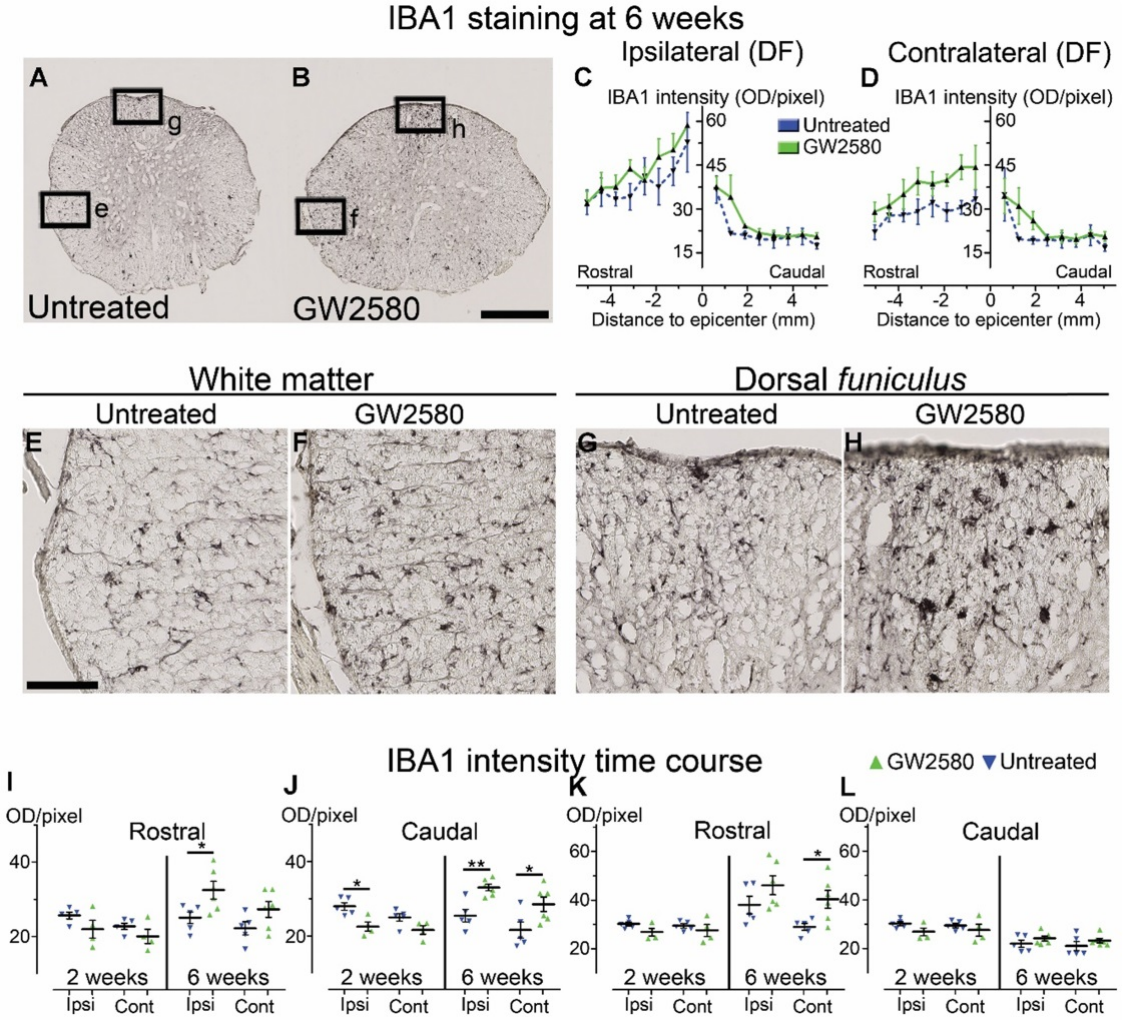
** Transient CSF1R blockade after lateral spinal cord hemisection in mice modulates microglial reactivity.** Bright-field micrographs showing IBA1-positive microglia at 6 weeks after SCI in untreated (**A**, **E** & **G**) and GW2580-treated (**B**, **F** & **H**) mice rostral to the lesion site. Higher magnifications (**E**-**H**) of black insets in **A**&**B**. Line curves display quantification of IBA1-immunoreactivity in the *dorsal funiculus* on the ipsilateral (**C**) and the contralateral (**D**) sides of the injured spinal cord along the rostro-caudal axis. Quantifications of IBA1-immunoreactivity in segments rostral (**I** & **K**) and caudal (**J** & **L**) to the lesion. Quantification in the white matter (excluding the *dorsal funiculus*) (**I** & **J**) and the *dorsal funiculus* (**K** & **L**) at 2 and 6-weeks following SCI. IBA1-immunoreactivity was quantified on ipsilateral and contralateral sides of the spinal cord. Scale bars: 500 µm (**A-B**), and 100 µm (**E**-**H**). Number of mice: 2 weeks n = 5 for untreated and n = 4 for treated; 6 weeks n = 6 for untreated and n = 6 for treated. Data are mean ± SEM per group. Student's unpaired t-test, *p < 0.05, **p < 0.01.

We then quantified microglial reactivity, using IBA1, a specific microglia marker, at 2 and 6 weeks after SCI on a 1 cm-perilesional segment of the spinal cord of untreated (**Figure 2A**) and GW2580 treated mice (**Figure 2B**). As we observed a differential microglia activation after SCI within the dorsal *funiculus* as compared to the overall white matter, we analyzed separately the dorsal *funiculus* (**Figure 2C-D**; **G-H** and** K-L**) and the white matter excluding the dorsal *funiculus*, (**Figure 2E-F**&**I-J**). Notably, analysis of IBA1 expression in the dorsal *funiculus* 6 weeks after SCI along the rostro-caudal axis revealed a higher microglia activation in the rostral segment on both ipsilateral (**Figure 2C**) and contralateral (**Figure 2D**) sides of the spinal cord as well as a constant higher microglia activation in the GW2580 group. Two weeks after lesion, in the white matter of the rostral segment, IBA1 expression was similar between GW2580-treated and untreated groups (**Figure 2I**) conversely to the ipsilateral side of the caudal segment where GW2580 treated mice presented a lower IBA1 expression than the control (**Figure 2J**, p<0.01). In the dorsal *funiculus*, 2 weeks after SCI, IBA1 expression was similar in both segments in the two groups (**Figure 2K-L**). Six weeks after lesion, the white matter of GW2580-treated mice displayed a higher IBA1 expression on the ipsilateral side of the spinal cord in the rostral segment (**Figure 2E-F**&**I**, p<0.01) and on both sides of the spinal cord in the caudal segment (**Figure 2J**, ipsilateral p<0.05, contralateral p<0.01). In the dorsal *funiculus*, IBA1 expression was also higher contralateral to the lesion in the rostral segment (**Figure 2G-H** & **K**), conversely to the caudal segment where microglial reactivity was similar in both groups (**Figure 2L**).

These data suggest that a transient 1-week inhibition of microglia proliferation in mice using GW2580, slightly reduces IBA1 expression in the white matter 2 weeks after SCI. This is followed by an overall increase in IBA1 expression 6 weeks after lesion.

### Transient post-injury GW2580 treatment in mice modifies outcomes on myelinated fibers

**Figure 3:**
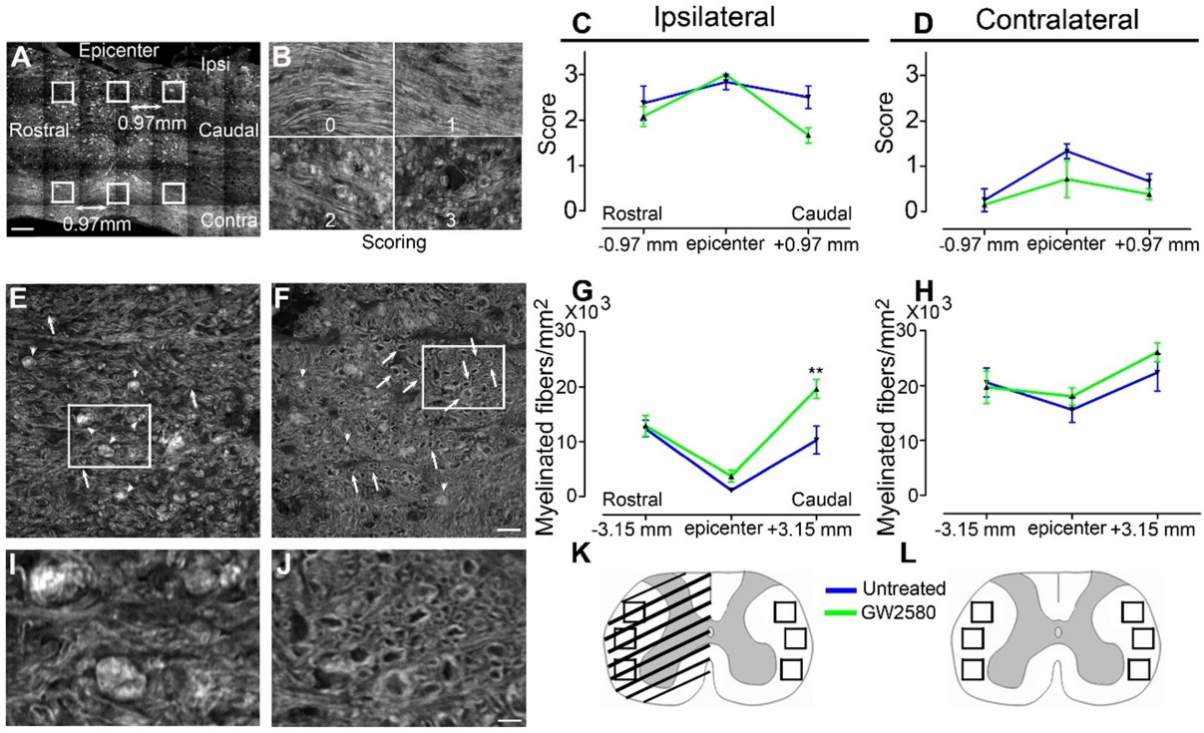
** Transient CSF1R blockade after SCI in mice modifies outcomes on myelinated fibers.** Sagittal CARS low resolution mosaic of a mouse spinal cord to indicate locations of the 6 images acquired per mouse (white boxes) used to score myelin morphology (**A**). Myelin scorings (**B**), normal white matter is associated with the score zero; scores 1 and 2 reflect an increasing occurrence of lipid debris and disorganized axonal arrangement and score 3 represents a complete loss of axonal alignment and major lipid debris. Myelin morphology scores quantified 6 weeks after SCI on sagittal sections of the spinal cord ipsilateral (**C**) and contralateral (**D**) to the lesion site. Representative CARS axial imaging of myelin after SCI in untreated (**E** & **I**) and GW2580-treated (**F** & **J**) mice. Quantification on axial sections of myelinated fibers/mm^2^ ipsilateral (**G**) and contralateral (**H**) to the lesion site 6 weeks after a lateral hemisection of the spinal cord in untreated and treated groups. Schematic spinal cord, boxes indicate locations of the 6 images acquired per mouse to quantify myelinated fibers density at the epicenter (**K**) and rostral and caudal to the lesion (**L**). In all graphs, results obtained by untreated mice are in blue and GW2580-treated mice in green. Scale bars: 500 µm (**A**); 20 µm (**B**); 20 µm (**E-F**), and 5 µm (**I** & **J**). Number of mice: n = 3 for untreated and treated groups for both axial and sagittal sections. Myelinated fibers: for each animal, 3 images per rostro-caudal location (-3.15 mm, epicenter and +3.15 mm) were quantified on both the ipsilateral and contralateral sides. Data are mean ± SEM per group. Student's unpaired t-test, *p < 0.05.

Microglial activation following SCI plays an important role in myelin breakdown and clearance 42. We thus investigated whether GW2580 treatment could affect outcomes on myelinated fibers using coherent anti-stokes Raman scattering (CARS) microscopy (**Figure 3**). Using a scoring method to assess label-free multiphoton myelin morphology 43, we first scored myelin degradation on sagittal spinal cord sections of treated and untreated mice (**Figure 3A-D**) at 6 weeks after SCI. We assessed myelin sheaths morphology on 3 locations in close vicinity of the lesion (lesion epicenter and 0.97mm rostral and caudal to the lesion, **Figure 3A**) on the ipsilateral and contralateral sides of the lesion site. Scores ranged from 0 (normal white matter) to 3 (loss of axonal alignment and predominant lipid debris (**Figure 3B**). No significant difference between groups was observed ipsilateral and contralateral to the lesion (**Figure 3C** & **D**). Using axial spinal cord sections (**Figure 3K**), we then quantified the density of intact myelinated fibers (**Figure 3E-H**, arrows in **E**&**F, I-J** zoom of boxes in **E** and **F**, respectively) at the injury epicenter and more distally from the lesion site (3.15mm rostral and caudal) in both treated and untreated animals at 6 weeks after SCI. Three distinct fields were analyzed in the lateral *funiculi* (**Figure 3K-L**) ipsilateral (**Figure 3G**) and contralateral (**Figure 3H**) to the lesion. GW2580 treatment induced a higher intact myelinated fibers density as compared to control ipsilateral to the lesion in the caudal segment (**Figure 3G**) but not contralateral to the lesion side (**Figure 3H**). Altogether, these results demonstrate that a transient GW2580-treatment after SCI in mice modifies outcomes on myelinated fibers following injury.

### Transient GW2580 treatment post-SCI in nonhuman primates reduces microglia proliferation

**Figure 4:**
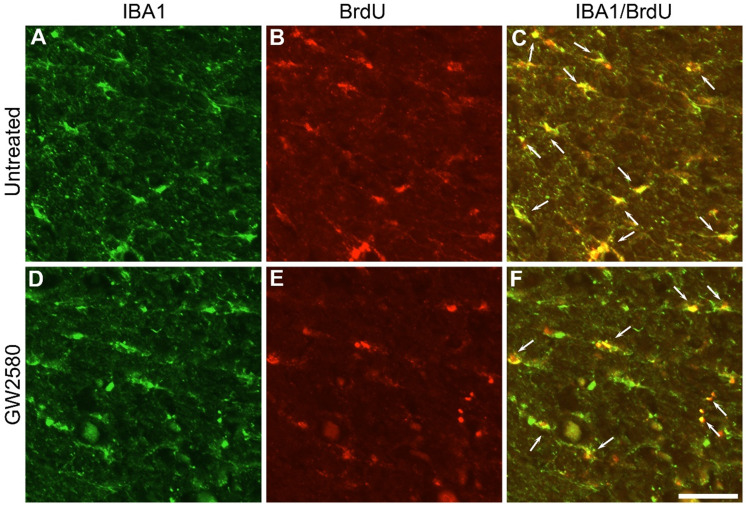
** Transient CSF1R blockade after lateral spinal cord hemisection in nonhuman primate decreases microglia proliferation.** Fluorescent micrographs of axial spinal cord sections from untreated (**A**-**C**) and GW2580-treated (**D-F**) *Microcebus murinus* at 1 week after SCI. All images were taken on the contralateral side 5 mm rostral to the lesion epicenter. IBA1 staining (**A** & **D**), BrdU staining (**B** & **E**) and merged (**C** & **F**). Arrows (**C** & **F**) indicate proliferative microglia (BrdU^+^/IBA1^+^). Scale bar: 50µm. Number of *Microcebus murinus analyzed*: n = 3 for untreated and n = 3 for treated animals.

In order to investigate whether oral GW2580 treatment would also induce beneficial effects on functional recovery after SCI in nonhuman primates, we first examined whether GW2580 also reduces microglia proliferation after lateral hemisection of the spinal cord in *Microcebus murinus*. Six male *Microcebus murinus* underwent a lateral hemisection of the spinal cord, three were untreated and three were treated with 7.2mg/day of GW2580 for 1 week. Animals were daily injected with BrdU during treatment and their spinal cords were analyzed. One week following SCI, microglia (IBA1^+^, **Figure 4A** & **D**), proliferating cells (BrdU^+^, **Figure 4B** & **E**) and proliferating microglia (IBA1^+^/BrdU^+^ cells, **Figure 4C** & **F**) were stained in untreated (**Figure 4A**-**C**) and GW2580-treated *Microcebus murinus* (**Figure 4D**-**F**). We observed an overall decrease in microglia proliferation in the GW2580-treated animal (**Figure 4F**, arrows) as compared to the non-treated one (**Figure 4C**, arrows). One week following SCI, proliferating cells (BrdU^+^ cells), microglia (IBA1^+^) and proliferating microglia (IBA1^+^/BrdU^+^ cells) were counted in GW2580-treated and untreated lemurs. For each animal, quantifications were done on 2 sections located 5 and 5.28 mm on the ipsilateral side rostral to the lesion site. No difference between GW2580-treated and untreated groups was observed in proliferating cells, indeed densities of 479±48 and 498±59 of BrdU^+^ cells per mm^2^ were counted, respectively. Similarly, microglia densities (IBA1^+^ cells) were not affected since 588±66 and 735±73 cells per mm^2^ were counted in the GW2580-treated and untreated groups respectively. Density of proliferative microglia (IBA1^+^/BrdU^+^ cells) in the GW2580-treated group was reduced as compared to the non-treated treated group since 230±23 and 300±49 cells/mm^2^ were counted, respectively. These findings demonstrate that GW2580 reduces microglial proliferation after SCI in nonhuman primates.

### Transient GW2580 treatment post-SCI in nonhuman primates improves motor recovery

**Figure 5:**
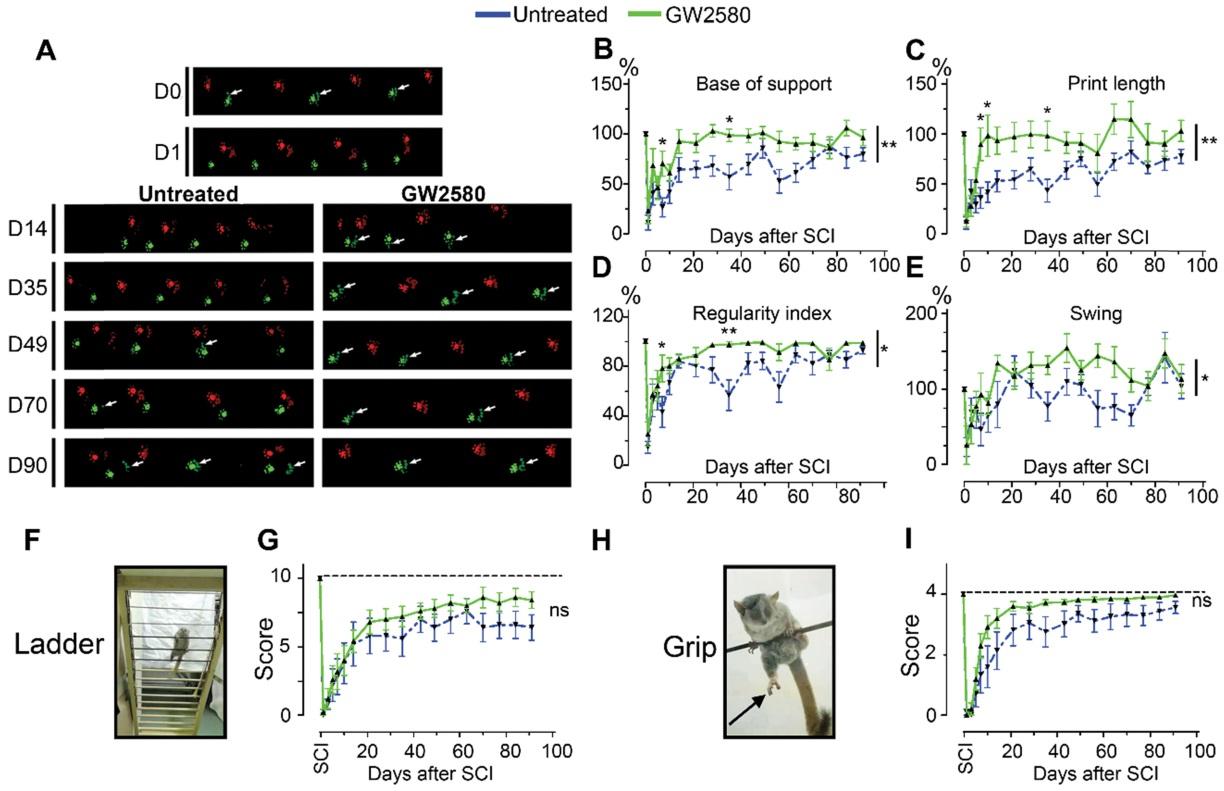
** Transient CSF1R blockade after lateral spinal cord hemisection in nonhuman primates improves motor function recovery.** Representative CatWalk™ runs of *Microcebus murinus* before (D0) and after (D1 to D90) lateral spinal cord hemisection (**A**). Front paws are represented in bright, hind paws in matte, contralateral paws in red and ipsilateral paws in green. White arrows point to the hind limb located on the injured side of the spinal cord. Line graphs displaying the base of support of the hind paws (p = 0.003, f = 10.27 and Df = 1) (**B**), the print length of the hind paw on the injured side of the spinal cord (p = 0.01, f = 7.66 and Df = 1) (**C**), the regularity index (p = 0.023, f = 5.80 and Df = 1) (**D**), and the swing phase of the hind limb located on the injured side of the spinal cord (p = 0.011, f = 7.31 and Df = 1) (**E**). Photographs of the ladder (**F**) and the bar (**H**) behavioral tests used to score the grip function of nonhuman primates. Arrow points the hind limb located on the injured side of the spinal cord. Line graphs displaying scores obtained with the ladder (p = 0.377, f = 0.88 and Df = 1) (**G**) and grip (p = 0.137, f = 2.74 and Df = 1) (**I**) tests. In all graphs, results for untreated nonhuman primates are in blue and GW2580-treated in green. Data are mean ± SEM per group. Two-Way ANOVA followed by Bonferroni post-hoc tests, *p < 0.05 and **p <0.01. p = pvalue; f = f-values and Df = degree of freedom. Number of injured *Microcebus murinus*: untreated n = 5 and GW2580-treated for 2 weeks n = 5.

We next investigated whether oral GW2580 treatment would also induce beneficial effects on functional recovery after lateral spinal cord hemisection in nonhuman primates. We thus performed a low thoracic (T12-L1) lateral hemisection of the spinal cord in 10 adult males, 5 were orally treated with GW2580, while 5 were untreated. The treatment dose was of 7.2 mg/day and treatment duration was extended for 2 weeks since microglial activation is delayed in nonhuman primates compared to rodents 44. Following SCI, all animals presented a hind limb monoplegia ipsilateral to the spinal cord lesion; only 1 animal developed a transient and incomplete deficit of the contralateral hind limb that spontaneously recovered within 72 hours. All animals survived the surgery, none developed bladder dysfunction, self-biting, cutaneous infection or inflammation and none presented body weight decrease except on days following SCI that is partly due to the 12 hours of fasting prior to anesthesia. Behavioral CatWalk™ assessments were conducted prior to surgery and from 1 day to 3 months after injury (**Figure 5A**). Pre-operative runs were similar in both groups (**Figure 5A**, D0). Immediately after injury, in both groups and for all parameters we observed a severe decrease in performance compared to pre-operative values (100%) (**Figure 5A-E**), as indicated by the absence of the print corresponding to the hind paw located on the ipsilateral side of the lesion (**Figure 5A**, arrows in D0 compared to D1)*.* Motor function recovery significantly improved in the treated group as compared to the untreated, as attested by the re-appearing of a consistent print of the hind paw located on the ipsilateral side of the lesion from 14- and 49-days post-injury in the GW2580-treated and untreated groups, respectively (**Figure 5A**, arrows). We selected 4 accurate criteria to quantify motor function recovery: 2 static parameters *i.e.*, the base of support of the hind paws (distance between hind paws) and the print length of the hind paw ipsilateral to the lesion and 2 dynamic parameters *i.e.*, the regularity index (inter-paw coordination), and the duration of the swing phase (the time without contact of a given paw to the glass plate in a step cycle). The base of support of the hind paws returned to pre-operative values (100%) at 14 days post-injury in the treated group, contrariwise to the untreated group that returned to normal value at 80 days after SCI (**Figure 5B**, 2-Way ANOVA **p <0.01). The print length of the ipsilateral hind paw returned to normal from day 10 in GW2580-treated animals contrariwise to the untreated group that did not returned to the pre-operative value through the 3 months post-lesion (**Figure 5C**, 2-Way ANOVA **p <0.01). The regularity index displayed an earlier (from 7 days post-injury) and enhanced recovery in the treated group as compared to the untreated group (**Figure 5D**, 2-Way ANOVA *p <0.05). Finally, recovery of the swing of the ipsilateral hind paw also occurred significantly earlier in the GW2580-treated group than in the control group (**Figure 5E**, 2-Way ANOVA *p <0.05). We then developed two additional tests *i.e.*, the ladder (**Figure 5F**), and the grip tests (**Figure 5H**) specifically for *Microcebus murinus* (see **Table 1** for scoring method). Scoring of the ipsilateral hind paw at the ladder test before injury was 10/10 for all; 24 hours after SCI animals all scored 0/10 (**Figure 5G**). Three months, after SCI treated animals displayed a score of 8/10 while remaining around 6/10 in the untreated group (**Figure 5G**). Similarly, the grip score was also discriminative since treated animals almost returned to a normal pre-operative score (score = 4) approximately 35 days after injury conversely to the untreated group that barely returned to normal values (**Figure 5I**). Taken together, these data demonstrate that a transient GW2580-treatment after SCI improves both static and dynamic parameters of motor function recovery in nonhuman primate.

### Transient GW2580-treatment after SCI in nonhuman primates participates in tissue reorganization and modifies outcomes on myelinated fibers

**Figure 6:**
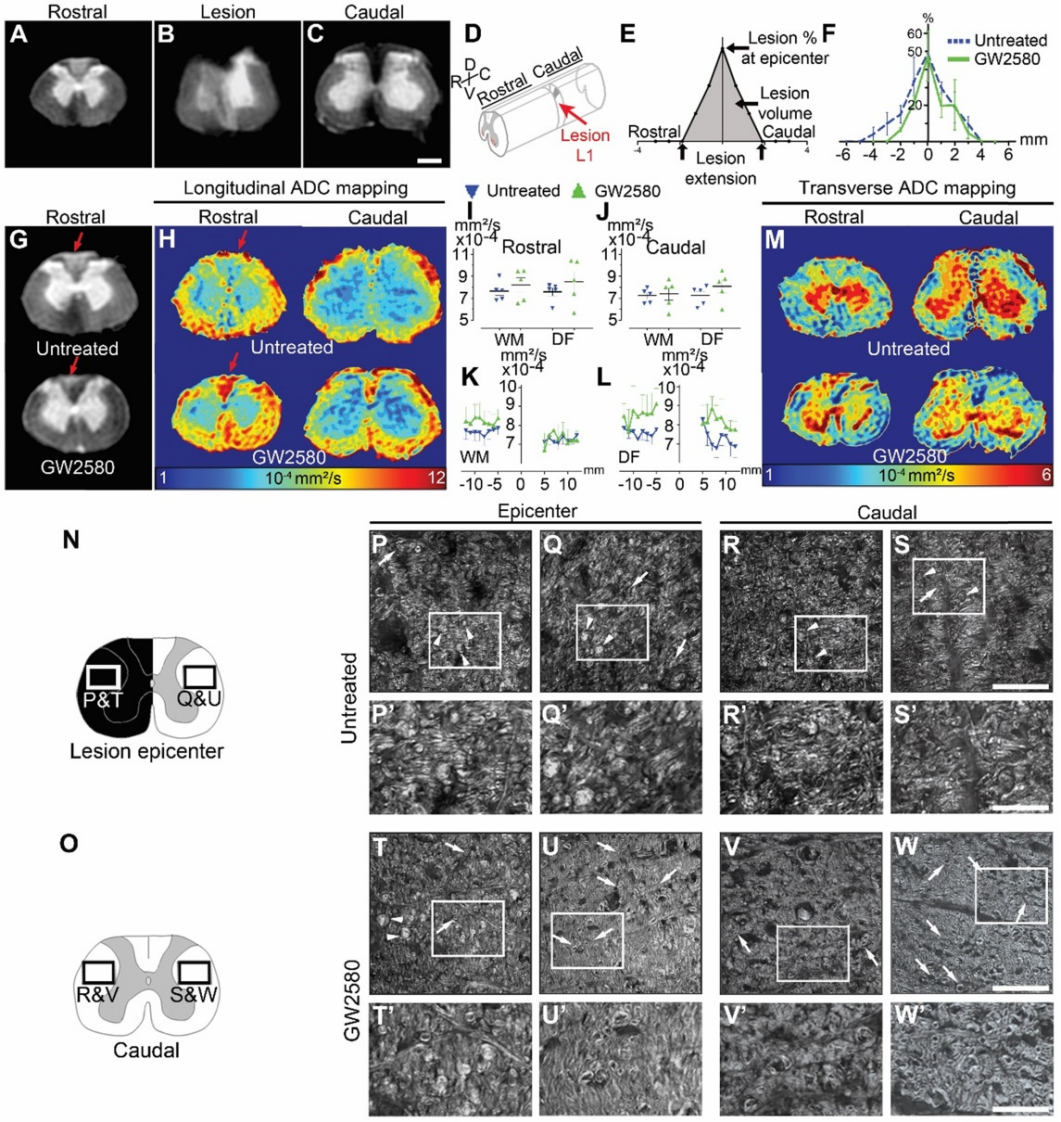
** GW2580 treatment after SCI in nonhuman primates preserves white matter ADC and modifies outcomes on myelinated fibers.** *Ex vivo* diffusion-weighted MRI rostral (**A**), within (**B**), and caudal (**C**) to the lesion 3 months after SCI in an untreated lemur. Schematic view of a T12-L1 lateral spinal cord hemisection (**D**). Schematic drawing of quantified parameters (**E**). Quantification 3 months following injury of the lesion percentage at the epicenter, the lesion extension and volume (area under the curve) (**F**). *Ex vivo* DW-MRI (**G**), longitudinal (**H**), and transverse (**M**) ADC mapping in treated and untreated animals. Red arrows in (**G&H**) indicate hyper-intense signal on both sides of the dorsal *funiculus* (DF) (untreated) and only on the hemisected side (GW2580). Longitudinal (**I**-**J**) diffusivities in the white matter and the DF. Quantifications were done rostral (**I**) and caudal (**J**) to the lesion epicenter. Quantification of LADC in the white matter (without DF) (**K**) and the DF (**L**) along the rostro-caudal axis. Schemes of the spinal cord at the lesion epicenter (**N**) and 2.1mm caudal to the lesion (**O**). CARS images (**P**-**W'**) taken in insets area presented in **N**&**O**. Myelin organization after SCI in untreated (**P**-**S'**) and GW2580-treated (**T**-**W'**) primates at the lesion epicenter (**P**&**P'**; **Q**&**Q'**; **T**&**T'** and **U-U'**) and caudal (**R**&**R'**;** S**&**S'**; **V**&**V'** and **W**&**W'**) to the lesion. Images ipsilateral (**P**&**P'**, **T**&**T'**, **R**&**R'**; and **V**&**V'**) and contralateral (**Q**&**Q'**; **U**&**U'**, **S**&**S'**; and **W**&**W'**) to the lesion. Insets in **P**-**S** and **T**-**W** correspond to higher magnifications in **P'**-**S'** and **T'**-**W'** respectively. Results for untreated nonhuman primates are in blue and GW2580-treated in green. Data are mean ± SEM per group. Student's unpaired t-test, *p < 0.05. Scale bars (**A-C&G**): 600µm; (**P**-**S** and **T**-**W**): 50 µm and (**P'**-**S'** and **T'**-**W'**): 20µm. Number of animals for MRI experiments: 5 untreated and 5 GW2580-treated and 1 animal in each group for CARS experiments.

To study the effect of GW2580 treatment on lesion size and spinal cord microstructure, in particular myelin, we used *ex vivo* diffusion-weighted magnetic resonance imaging (DW-MRI). White and grey matters as well as the lesion site were clearly identifiable (**Figure 6A**-**C**). The lesion epicenter was identified as the section with the highest percentage of damaged tissues (**Figure 6B**, **D**&**E**). Three months after lesion, percentage of damaged tissues at the injury epicenter (50% in both groups), lesion extension (5.6+/-0.5 and 5.4+/-0.4mm in the untreated and GW2580 treated group, respectively) and lesion volume (AUC 168+/-22.5 and 120.3+/-35.6 in the untreated and GW2580 treated group, respectively) were similar in both groups (**Figure 6E**&**F**). Rostral to the lesion, we observed a hypersignal in both sides of the dorsal *funiculus* (DF) in the untreated group and only on the ipsilateral side of the lesion in GW2580-treated animals (**Figure 6G**, red arrows). We thus quantified longitudinal (**Figure 6H-L**) and transverse (**Figure 6M**) diffusivities on a 2 cm-spinal cord segment centered on the lesion site separately in the white matter (WM) excluding the *dorsal funiculus* and the DF. Longitudinal apparent diffusion coefficient (LADC) was similar in both the WM and the DF of GW2580-treated as compared to untreated animals rostral and caudal to the lesion (**Figure 6I-J**). However, an analysis of LADC in the WM (without DF, **Figure 6K**) and the DF (**Figure 6L**) along the rostro-caudal axis highlighted that the GW2580-treated LADC curve was always above the non-treated curve. No difference was detected between groups for transversal apparent diffusion coefficient (TADC, data not shown).

We further assessed the effect of GW2580-treatment on SCI-induced demyelination using Luxol fast blue and neutral red staining on the same spinal cord 1-cm segment (**Supplementary Figure 1**). Quantification of myelin damage in the spinal cord using Luxol highlighted around 34% reduction, that however did not reach statistical significance, in the lesion percentage at the injury epicenter in the GW2580-treated group (44.45±8.7 and 29.15±4.8% in untreated and treated groups, respectively, **Supplementary Figure 1C-D**). No difference between groups in the spared white matter rostral (**Supplementary Figure 1A-B**) and caudal (**Supplementary Figure 1E-F**) to the lesion epicenter was seen on both ipsilateral and contralateral sides. To further investigate if GW2580 treatment could act on the overall protection of myelin break-down, we used CARS on axial sections of the spinal cord of untreated (**Figure 6P-S'**) and GW2580-treated animals (**Figure 6T-W'**) taken at the lesion epicenter (**Figure 6N**,** P-Q**; **P'-Q'**, **T-U** and** T'-U'**) and 2.1 mm caudal to the lesion (**Figure 6O**,** R-S**;** R'-S'**,** V-W** and **V'-W'**) on both ipsilateral (**Figure 6P**&**P'**and** T**&**T'**; **R**&**R'** and** V**&**V'**) and contralateral sides (**Figure 6Q**&**Q'**and** U**&**U'**; **S**&**S'** and** W**&**W'**) of the lesion. Intact myelin sheaths were almost absent in all sections of untreated animals (**Figure 6P-S**, arrows and **P'-S'**). Conversely, well-distinguishable intact myelin sheaths were seen in the contralateral side of the spinal cord of treated animals (**Figure 6U**&**U'**, and **W**&**W**', arrows) and to a lesser extent on the ipsilateral side of the lesion (**Figure 6T**&**T'** and** V**&**V**', arrows). Lipid accumulation, most likely formed by myelin debris, were also regularly observed in the spinal cord of untreated *Microcebus murinus* (**Figure 6P** & **Q** and** P'**&**Q'**; **R**&**S** and **R'**&**S'** arrowheads) contrariwise to GW2580-treated animals (**Figure 6T** & **U** and** T'**&**U'**; **V** & **W** and **V'** & **W'** arrowheads). To deepen our analyses on myelin, we quantified the density of myelinated fibers using fluoromyelin staining in 4 animals per group (**Supplementary Figure 2**). We analyzed lateral *funiculi* in one section per animal located 1.68 mm caudal to the lesion epicenter. On both the ipsilateral (**Supplementary Figure 2A-C**) and the contralateral (**Supplementary Figure 2D-F**) side of the lesion no statistically significant difference was seen between untreated and GW2580-treated groups. However, density comparison between ipsilateral and contralateral sides in the treated group (**Supplementary Figure 2B E** & **H**) highlighted, conversely to the untreated group (**Supplementary Figure 2A, D** & **G**), a higher density contralaterally than ipsilaterally.

Thus, a transient GW2580 treatment post-SCI in nonhuman primates evokes an overall limitation of secondary damage reflected by modification in outcomes on myelinated fibers.

### Microglia reactivity returns to baseline 3 months after GW2580 treatment in nonhuman primates

**Figure 7:**
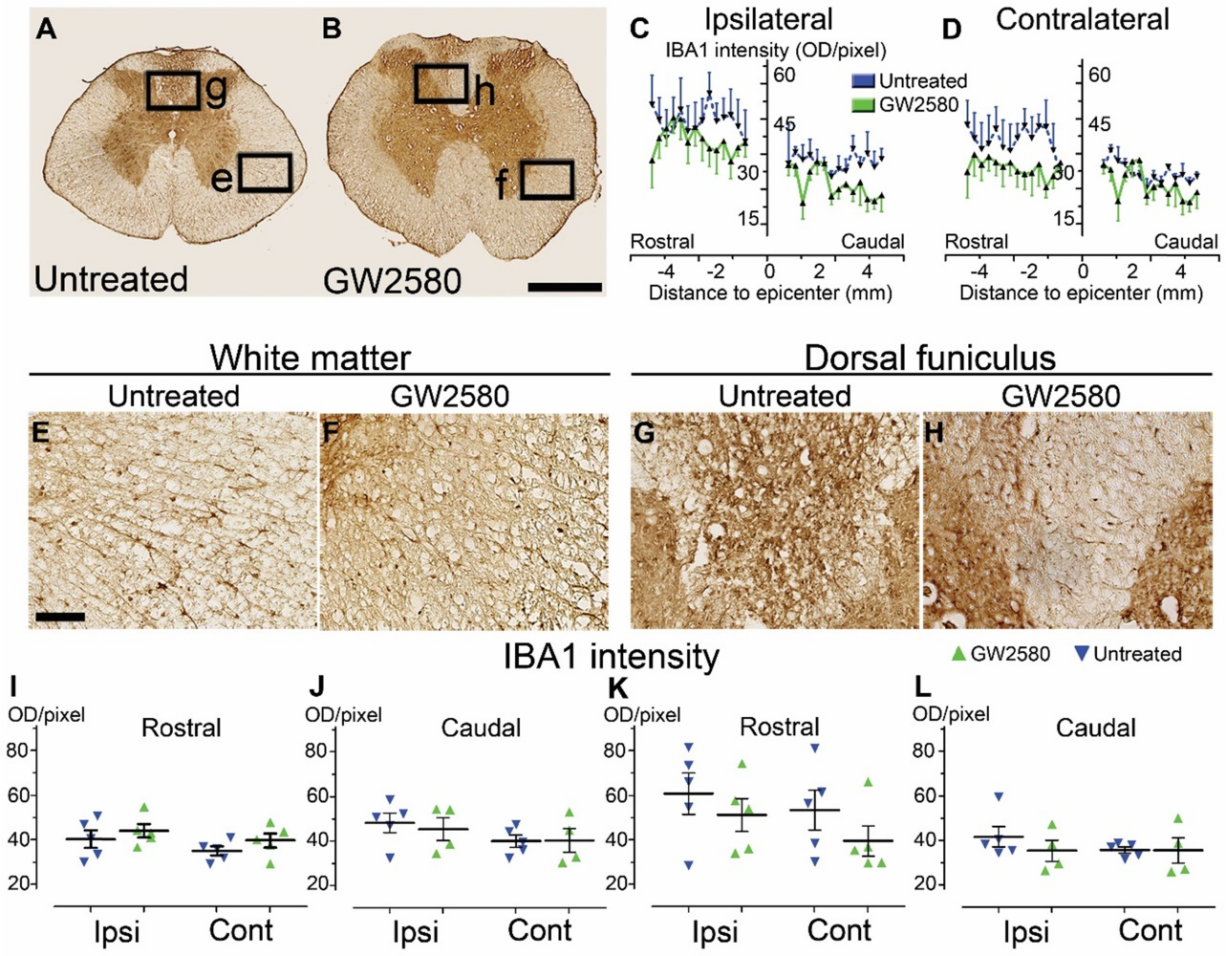
** Transient CSF1R blockade after lateral spinal cord hemisection in nonhuman primates does not affect microglial reactivity in the long term.** Bright-field micrographs showing IBA1-positive microglia after SCI in untreated (**A**, **E** & **G**) and GW2580-treated (**B**, **F** & **H**) nonhuman primates rostral to the lesion site 3 months after SCI. Higher magnifications (**E**-**H**) of black insets in **A** & **B**. Line curves display quantification of IBA1-immunoreactivity in the dorsal *funiculus* on the ipsilateral (**C**) and the contralateral (**D**) sides of the injured spinal cord along the rostro-caudal axis. Quantifications of IBA1-immunoreactivity in segments rostral (**I** & **K**) and caudal (**J** & **L**) to the lesion. Quantification in the white matter (excluding the dorsal *funiculus*) (**I** & **J**) and the dorsal *funiculus* (**K**&**L**) at 3-months following SCI. IBA1-immunoreactivity was quantified on ipsilateral and contralateral sides of the spinal cord (**I**-**L**). Results for untreated nonhuman primates are in blue and GW2580-treated in green. Data are mean ± SEM per group. Student's unpaired t-test was used. Scale bars (**A** & **B**): 500 µm; (**E** & **H**): 100 µm. At least 40 sections (centered on the lesion site) per animal at 210µm intervals were analyzed. Number of *Microcebus murinus*: injured & untreated n = 5, injured & GW2580-treated for 2 weeks n = 5.

Similar to mice, we quantified microglial reactivity at 3 months after SCI on a 1cm-perilesional segment of the spinal cord using IBA1 (**Figure 7**). We did not observe a differential activation of microglial cells in between untreated (**Figure 7A, E** & **G**) and GW2580-treated nonhuman primates (**Figure 7B, F** & **H**), neither within the white matter (excluding the dorsal *funiculus*) (**Figure 7I** & **J**) nor in the dorsal *funiculus* (**Figure 7K** & **L**). However, in the dorsal *funiculus* rostral to the lesion, microglial reactivity was overall limited to the ipsilateral side of the lesion in GW2580-treated nonhuman primates (**Figure 7B**,** C** & **D**,** H**) while it usually spreads in the entire *funiculus* in the untreated group (**Figure 7A**,** C** & **D, G**).

These data suggest that a transient inhibition of microglia proliferation using GW2580, does not affects overall IBA1 expression in the long term after SCI in nonhuman primates.

### GW2580 treatment after SCI does not modify muscle fibers surface and neuromuscular junction density in nonhuman primates

**Figure 8:**
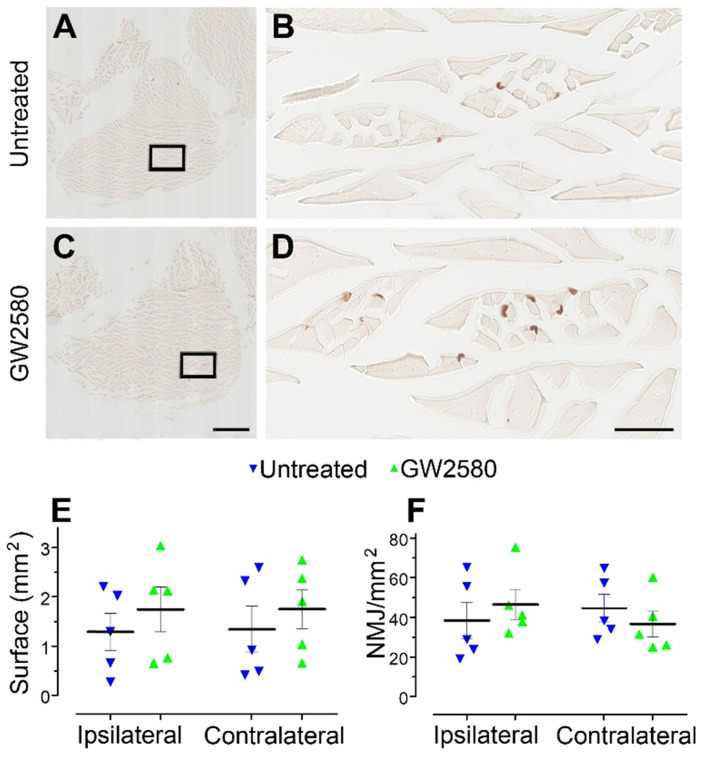
** Transient CSF1R blockade after lateral spinal cord hemisection in nonhuman primates does not affect muscle surface and neuromuscular junction density.** Bright-field micrographs showing *gastrocnemius-soleus-plantaris* muscle complex of the hind limb located on the ipsilateral side of the spinal cord lesion in untreated (**A**) and GW2580-treated (**C**) *Microcebus murinus*. Black boxes in **A** & **C** correspond to higher magnification taken within the *gastrocnemius* muscle and presented in **B** & **D**, respectively. Graphs displaying quantitative assessments of the *gastrocnemius* muscle fiber surface area (**E**) and the density of neuromuscular junctions (**F**). In all graphs, results for untreated nonhuman primates are in blue and GW2580-treated are in green. Data are mean ± SEM per group. Student's unpaired t-test was used. Scale bars (**A** & **C**): 1 mm, (**B** & **D**): 100 µm. At least 20 sections per animal throughout the *gastrocnemius* muscle at 16 µm intervals were analyzed. Number of *Microcebus murinus*: injured & untreated n = 5, injured & GW2580-treated for 2 weeks n = 5.

Improvement in motor function recovery following SCI induced by GW2580 treatment suggests a better preservation of the skeletal hind limb muscles. We thus analyzed in both groups muscle fiber surface and the neuromuscular junctions (NMJ) density in the *gastrocnemius* of both hind limbs (**Figure 8A-D**). Overall, muscle fiber surface (**Figure 8E**) and NMJ density (**Figure 8F**) were similar in *gastrocnemius* located on the ipsilateral and the contralateral sides of the spinal cord lesion in both untreated and treated groups. Comparison between groups did not identify significant difference for both parameters. Therefore, GW2580 does not induce quantifiable difference between the ipsilateral and the contralateral muscle fiber surface and NMJ density of *gastrocnemius* three months after SCI in nonhuman primates.

### Identification of transcriptional microglial changes induced by GW2580-treatment after SCI in mice

**Figure 9:**
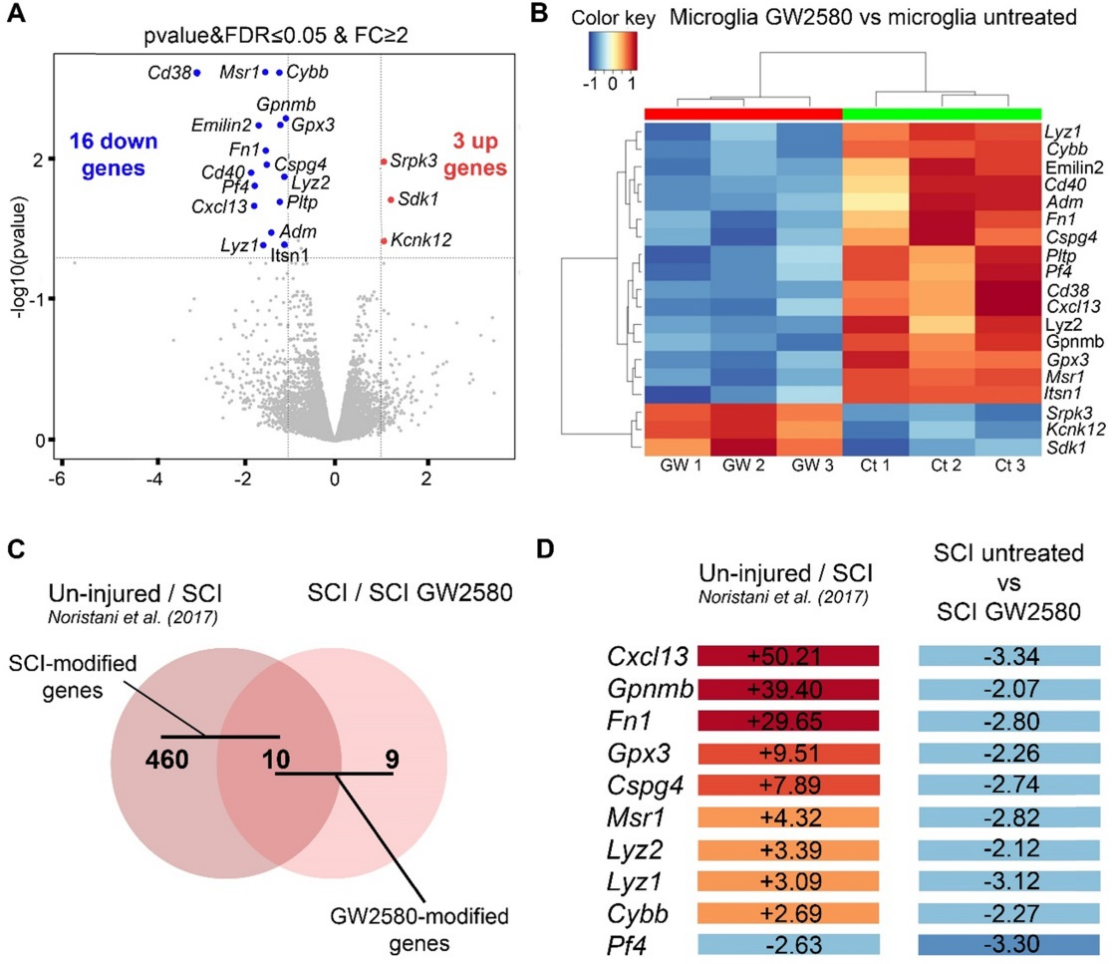
** Transient 1-week CSF1R blockade after SCI in mice induces transcriptional modification in microglia.** RNA-seq analysis of FACS-isolated microglia from pooled (at least 2 animals and 16.000 cells) 1cm-spinal cord segments centered on the lesion site of SCI untreated and treated mice at 1 week after injury (end of the treatment) (**A**-**B**). We selected the same stringent cutoff as in our previous study [Fold Change (FC)≥2 and p-value with false discovery rate (FDR) ≤0.05] 7. Volcano plot (**A**). Heat map (**B**). *In silico* differential expression analysis (**C**-**D**). Comparison of the list of genes deregulated by the injury [identified in our previous study using the same parameters: male CX3CR1^+/eGFP^ mice aged of 3 months, lateral hemisection of the spinal cord at T9 level, analysis of DE genes in microglia in a 1-cm segment centered on the lesion 1 week after injury (uninjured/SCI) 7) with the list of genes deregulated by GW2580 treatment in SC-injured mice (SCI-untreated */* SCI-GW2580). Venn diagram (**C**). Fold changes of the 10 genes commonly deregulated in the comparison between (un-injured/SCI) and (SCI-untreated / SCI GW2580) (**D**).

Finally, to initiate investigations on molecular mechanisms induced by a transient post-SCI GW2580-treatment we took advantage of the CX3CR1^+/eGFP^ transgenic mice that express eGFP in microglia and peripheral monocytes. We combined fluorescence-activated cell sorting and RNA-Seq analysis. To focus on microglia, we isolated only eGFP^high^ positive cells from treated and untreated CX3CR1^+/eGFP^ spinal cord injured mice. Indeed, we have previously shown in this injury model that 1-week after lesion eGFP^high^ expressing cells located in a 1 cm segment centered on the injury site are CD11^+^/LY6C^neg/low^/GFP^high^ and thus mostly correspond to microglia without contamination of infiltrating monocytes 7. Transcriptomic analyses were done at the end of the treatment (1 week after injury) on microglia from pooled 1cm-spinal cord segments centered on the lesion site, as we previously described 7. We applied the same stringent cutoff as in our previous study [Fold Change (FC)≥2 and p-value with false discovery rate (FDR) ≤0.05] 7. We found 19 differentially expressed (DE) genes; 16 and 3 genes were down and up-regulated, respectively in the GW2580-treated groups as compared to the untreated control (**Figure 9A**-**B** and **Supplementary Table 1**). Thus, microglia from depleted animals clearly displayed a higher number of down than up-regulated genes. Unbiased hierarchical clustering revealed a strong reproducibility in DE genes among independent biological replicates (**Figure 9B**). Notably, down regulated genes belong to processes such as regulation of cell proliferation and cell migration [*chondroitin sulfate proteoglycan 4* (*Cspg4*), *macrophage scavenger receptor 1* (*Msr1*), *glycoprotein transmembrane* (*Gpnmb*), *adrenomedullin* (*Adm*), and *fibronectin 1* (*Fn1*)], inflammatory response [*platelet factor 4* (*Pf4*), *cytochrome b-245* (*Cybb*)], immune response [*lysozyme* (*Lyz1 and 2*), *Cd40* (*nfrsf5*), *chemokine (C-X-C motif) ligand 13* (*Cxcl13*)] (**Supplementary Table 1**). Remarkably, Gene Ontology (GO) enrichment analysis of down-regulated genes ranked as top molecular function “CXCR3 chemokine receptor binding (2 DE genes; p-value with FDR: 7.314E-04) and ranked as top processes the inflammatory response (8 DE genes; p-value with FDR 5.588 10E-05, **Supplementary table 2**).

Previously, we have identified injury induced DE genes in microglia in a 1-cm segment surrounding a T9 spinal cord lateral hemisection in CX3CR1^+/eGFP^ mice 1 week after injury 7. To determine whether DE SCI-induced genes were modified by GW2580 treatment, we compared the list of genes deregulated by the injury (uninjured */* SCI) 7 with the list of genes deregulated by GW2580 treatment in SC-injured mice (SCI-untreated */* SCI-GW2580) (**Figure 9C-D**). Out of the 470 DE genes by SCI in microglia, 10 were changed by GW2580. Ninety percent (9 out of 10) of these genes were down-regulated by GW2580 treatment. Strikingly, *Cxcl1*3 the most up-regulated gene by SCI (+50.21) is involved in immune response process and 4 out of these 9 genes were involved in cell proliferation and cell migration (*Cspg4*, *Gpnmb, Msr1*, and *Fn1*).

Taken together, these data demonstrate that a transient GW2580 treatment not only deplete microglia proliferation, but also reduces their inflammatory response that may account for improved functional recovery following SCI.

## Discussion

Microglia and macrophages are recognized as pivotal players in SCI pathophysiology 4, 19, 45-47. They play dual beneficial and detrimental roles after SCI that most likely depend on the kinetics of their responses, including proliferation 3, 4, 19. Our findings suggest that a transient depletion of microglia proliferation immediately after SCI improves functional recovery in mice and nonhuman primates.

Following SCI, unspecific modulation of anti- or pro-inflammatory responses were often ineffective, if not detrimental. Modulating microglia should thus be done with caution to limit additional risks including induction of systemic diseases already triggered by the SCI-induced disruption of the neural-immune communication (for review see 45). We have therefore chosen to transiently inhibit microglial proliferation shortly after SCI.

We first extended our previous results that demonstrated that a pre-and post-SCI GW2580-treatment specifically reduces microglial proliferation 23 to a more translational paradigm *i.e.* a short term post-injury treatment. In both mice and nonhuman primates, microglia were still present after 1 week of treatment and microglia proliferation was reduced. *In vitro*, long-term exposure to BrdU reduces the proliferating activity of lung cancer cells 48 and a single low-dose of BrdU induces antiproliferative effect in murine stem and progenitor cells 49. *In vivo*, repeated exposure to BrdU in a short period of time (4 injections/day, 50-100 mg/kg) also reduces primary neural stem/progenitor cells proliferation 2 hours after the last injection 50 and repeated long-term BrdU injections (3 days/week, 14 weeks, 200 mg/day) inhibit proliferation of alveolar epithelial and mesenchymal cells in mice 4. In all these studies the dose and/or the BrdU exposure duration differ from our experiments. We cannot exclude an antiproliferative effect of BrdU on microglia, however all injected animals (both species) were used only for proliferation assessment and not for behavioral analysis. We can thus exclude a concomitant effect of BrdU and GW2580 on functional recovery.

Secondly, we highlighted that a transient GW2580 treatment initiated immediately after SCI enhanced motor recovery in mice and nonhuman primates. Interestingly, improved motor function recovery seems greater in nonhuman primates than in rodents. Enhanced recovery in GW2580-treated lemur started as early as 10-15 days after injury and several parameters eventually returned to pre-injury values. This is consistent with a previous study that also reported better functional recovery in primates than rodents after a lateralized SCI 32. Besides, we observed in the white matter a trend of higher DW-MRI diffusivities along the rostro-caudal axis in treated, as compared to untreated, lemur that reflect tissue reorganization and may result from modification of injury outcomes on myelin in treated *Microcebus murinus* 3 months after injury (for review see 51, 52). Even if yet not frequently used in SCI 53, 54, Raman spectroscopy can accurately detect SCI-associated microglial inflammation and myelin degradation 43, 55. Indeed, combination of endogenous two-photon fluorescence and CARS imaging highlighted the presence of lipid debris in SCI-induced inflammatory region thus providing an indicator of activated microglia/macrophages following engulfment of myelin debris 56. The transitory inhibition of microglia proliferation also led to a decreased presence of lipid debris in mice and nonhuman primate. Lateral spinal cord hemisection results in severed axons that will undergo Wallerian degeneration ipsilateral and caudal to the lesion. The higher density of intact myelin sheaths observed caudally in treated mice 6 weeks after injury may thus reflect slower myelin clearance of the damage axons resulting from GW2580-induced delayed phagocytosis due to fewer microglia. Though, in *Microcebus murinus*, 3 months after the lesion we observed, caudal to the lesion, a higher density of myelin sheaths on the contralateral side as compared to the ipsilateral side only in treated animals. This may evoke a limitation of diffuse secondary damage, that in turns participate in better functional recovery.

Our results support recent findings showing that haploinsufficiency of sorting nexin 27 (*Snx27*) in mice, an endosome-associated cargo adaptor, that amongst other functions suppresses microglia/macrophages proliferation, improved motor recovery following SCI 57. Snx27 deficiency additionally reduces apoptotic neuronal death. Our findings also complement previous work that targeted microglia/monocytes in the context of SCI. First, a selective depletion of a subset of infiltrating Ly6C^+^(Gr1^+^)CCR2^+^ monocytes deteriorated motor recovery following SCI in mice 58. Second, using CCR2 null mice, it had been shown that stopping the crosstalk between resident microglia and monocyte derived macrophages participates in long-term microglial activation, greater myelin loss that eventually worsen motor function after SCI 5. Third, complete microglia depletion altered glial scar formation, decreased immune infiltration, neuronal and oligodendrocytes survival associated with impaired motor recovery following SCI in mice 19, 21. This difference as compared to our findings may result from the specific subpopulation of microglia (*i.e.,* only proliferative microglia) that is inhibited by GW2580 22, 23. Even if CSF1R is principally expressed by microglia in the intact CNS 59-61, we cannot exclude off-target effects of GW2580 treatment affecting neurons that also express CSF1R 62 or infiltrating macrophages (for review see 16). However, SCI induces a sevenfold greater microglia proliferation as compared to infiltrating macrophages 5. Conversely, *in vivo,* evidence of a neuroprotective role of proliferating microglia that may serve as an endogenous pool of neurotrophic molecules such as IGF-1 had been reported in cerebral ischemia 63. The difference may result from the heterogeneity and region-specific differences in microglial response 64.

In mice, transient inhibition of microglia proliferation induced a reduction of IBA1 expression 2 weeks after SCI followed by an overall increase in its expression 6 weeks after lesion that may correspond to a transient microglial over-repopulation. Likewise, in *Microcebus murinus*, microglia reactivity that returned to baseline 3 months after lesion may reflect microglial repopulation. Renewal of microglia is observed in physiological condition in mice 65-67 and humans 68. In pathological conditions, a clonal microglial expansion is reported in mice 66, 67 and in *Macaca fuscata* 69. Strikingly, microglial replenishment after traumatic brain injury in mice following short term PLX5622-induced depletion stimulates neurogenesis and decrease learning deficits 15, corroborating that transient microglia depletion early after traumatism is beneficial. However, understanding the exact kinetic and extend of microglia proliferation following SCI would raise the possibility of modulating their proliferation in more chronic phases.

Finally, our transcriptomic analyses of microglia highlighted that post-injury GW2580-treatment down-regulated the expression of genes involved in cell proliferation, cell migration and inflammatory response, which is consistent with an inhibition of microglia proliferation/activation. We also emphasized that GW2580-treatment after injury reverse the up-regulation of 9 genes induced by SCI. Notably, 4 out of the 9 genes are involved in cell proliferation and cell migration, robustly confirming that GW2580-treatment inhibits SCI-induced microglia proliferation/activation. Interestingly, *Cxcl1*3 that is involved in inflammatory response in CNS diseases including multiple sclerosis and progressive myoclonus epilepsy of Unverricht-Lundborg type 70-72 was strongly up-regulated by SCI (FC = +50.21) and decreased by GW2580 treatment (FC = -3.34), suggesting that GW2580 may inhibit neuroinflammation through CXCL13-mediated signaling pathway in SCI. This is consistent with reports showing in rat spinal cord ischemia-reperfusion that inhibition of microglia activation and CXCL13/CXCR5 axis induced neurological and histological improvement 73-75. Chondroitin sulfate proteoglycans (CSPG) are extracellular matrix (ECM) molecules that have been recognized to limit axonal growth after CNS injury. Here we highlight that *Cspg4* that encodes nerve/glia antigen 2 (NG2) thatis strongly up-regulated by SCI (FC = +7.89) 7 is decreased by GW2580 treatment (FC = -2.74). This is consistent with previous studies showing that following SCI reactive macrophages and oligodendrocyte progenitor express NG2 76 and that after brain injury, activated microglia express NG2 at least within the first week 77. Further investigations of the role of these genes in microglia proliferation will help to better understand molecular mechanisms induced by GW2580-treatment.

In conclusion, we show that a transient post-injury oral administration of GW2580, inhibiting microglia proliferation, promotes motor functional recovery and modulates tissue structure following SCI in rodents and nonhuman primates. Beneficial effects of GW2580-treatment on motor recovery seem greater in *Microcebus murinus* than in mice, pointing to the key role of nonhuman primates as critical SCI models to further promote translational research.

## Figures and Tables

**Table 1:** Scoring of the ladder test for *Microcebus murinus*

Percentage of successful bars climbed on total bar number (per given hind/paw)	Scoring of the movement of the paw	Scoring of the capacity to grip the bar
0	0	0
0-24	1	1
25-50	2	2
51-74	3	3
75-99	4	4
100% (normal)	5	5

## References

[1] Kumar R, Lim J, Mekary RA, Rattani A, Dewan MC, Sharif SY (2018). Traumatic spinal injury: Global epidemiology and worldwide volume. World neurosurg.

[2] Gaudet AD, Fonken LK (2018). Glial cells shape pathology and repair after spinal cord injury. Neurotherapeutics.

[3] David S, Kroner A (2011). Repertoire of microglial and macrophage responses after spinal cord injury. Nat Rev Neurosci.

[4] David S, Kroner A, Greenhalgh AD, Zarruk JG, Lopez-Vales R (2018). Myeloid cell responses after spinal cord injury. J of neuroimmunol.

[5] Greenhalgh AD, Zarruk JG, Healy LM, Baskar Jesudasan SJ, Jhelum P, Salmon CK (2018). Peripherally derived macrophages modulate microglial function to reduce inflammation after CNS injury. PLoS Biol.

[6] Greenhalgh AD, David S (2014). Differences in the phagocytic response of microglia and peripheral macrophages after spinal cord injury and its effects on cell death. J Neurosci.

[7] Noristani HN, Gerber YN, Sabourin J-C, Le Corre M, Lonjon N, Mestre-Frances N (2017). RNA-Seq analysis of microglia reveals time-dependent activation of specific genetic programs following spinal cord injury. Front Mol Neurosci.

[8] Chitu V, Gokhan S, Nandi S, Mehler MF, Stanley ER (2016). Emerging roles for CSF-1 receptor and its ligands in the nervous system. Trends Neurosci.

[9] Elmore MR, Lee RJ, West BL, Green KN (2015). Characterizing newly repopulated microglia in the adult mouse: impacts on animal behavior, cell morphology, and neuroinflammation. PLoS One.

[10] Elmore MR, Najafi AR, Koike MA, Dagher NN, Spangenberg EE, Rice RA (2014). Colony-stimulating factor 1 receptor signaling is necessary for microglia viability, unmasking a microglia progenitor cell in the adult brain. Neuron.

[11] Beckmann N, Giorgetti E, Neuhaus A, Zurbruegg S, Accart N, Smith P (2018). Brain region-specific enhancement of remyelination and prevention of demyelination by the CSF1R kinase inhibitor BLZ945. Acta Neuropathol Commun.

[12] Dagher NN, Najafi AR, Kayala KM, Elmore MR, White TE, Medeiros R (2015). Colony-stimulating factor 1 receptor inhibition prevents microglial plaque association and improves cognition in 3xTg-AD mice. J Neuroinflammation.

[13] Hou B, Jiang C, Wang D, Wang G, Wang Z, Zhu M (2020). Pharmacological targeting of CSF1R inhibits microglial proliferation and aggravates the progression of cerebral ischemic pathology. Front Cell Neurosci.

[14] Spiller KJ, Restrepo CR, Khan T, Dominique MA, Fang TC, Canter RG (2018). Microglia-mediated recovery from ALS-relevant motor neuron degeneration in a mouse model of TDP-43 proteinopathy. Nat Neurosci.

[15] Willis EF, MacDonald KPA, Nguyen QH, Garrido AL, Gillespie ER, Harley SBR (2020). Repopulating microglia promote brain repair in an IL-6-dependent manner. Cell.

[16] Green KN, Crapser JD, Hohsfield LA (2020). To Kill a microglia: A case for CSF1R inhibitors. Trends Immunol.

[17] Wieghofer P, Knobeloch KP, Prinz M (2015). Genetic targeting of microglia. Glia.

[18] Henry RJ, Ritzel RM, Barrett JP, Doran SJ, Jiao Y, Leach JB (2020). Microglial depletion with CSF1R inhibitor during chronic phase of experimental traumatic brain injury reduces Neurodegeneration and Neurological Deficits. J Neurosci.

[19] Bellver-Landete V, Bretheau F, Mailhot B, Vallieres N, Lessard M, Janelle ME (2019). Microglia are an essential component of the neuroprotective scar that forms after spinal cord injury. Nat Comm.

[20] Li Y, Ritzel RM, Khan N, Cao T, He J, Lei Z (2020). Delayed microglial depletion after spinal cord injury reduces chronic inflammation and neurodegeneration in the brain and improves neurological recovery in male mice. Theranostics.

[21] Fu H, Zhao Y, Hu D, Wang S, Yu T, Zhang L (2020). Depletion of microglia exacerbates injury and impairs function recovery after spinal cord injury in mice. Cell Death Dis.

[22] Conway JG, McDonald B, Parham J, Keith B, Rusnak DW, Shaw E (2005). Inhibition of colony-stimulating-factor-1 signaling *in vivo* with the orally bioavailable cFMS kinase inhibitor GW2580. Proc Natl Acad Sci U S A.

[23] Gerber YN, Saint-Martin GP, Bringuier CM, Bartolami S, Goze-Bac C, Noristani HN (2018). CSF1R inhibition reduces microglia proliferation, promotes tissue preservation and improves motor recovery after spinal cord injury. Front Cell Neurosci.

[24] Crespo O, Kang SC, Daneman R, Lindstrom TM, Ho PP, Sobel RA (2011). Tyrosine kinase inhibitors ameliorate autoimmune encephalomyelitis in a mouse model of multiple sclerosis. J Clin Immunol.

[25] Gomez-Nicola D, Fransen NL, Suzzi S, Perry VH (2013). Regulation of microglial proliferation during chronic neurodegeneration. J Neurosci.

[26] De Lucia C, Rinchon A, Olmos-Alonso A, Riecken K, Fehse B, Boche D (2016). Microglia regulate hippocampal neurogenesis during chronic neurodegeneration. Brain Behav Immun.

[27] Olmos-Alonso A, Schetters ST, Sri S, Askew K, Mancuso R, Vargas-Caballero M (2016). Pharmacological targeting of CSF1R inhibits microglial proliferation and prevents the progression of Alzheimer's-like pathology. Brain.

[28] Neal ML, Fleming SM, Budge KM, Boyle AM, Kim C, Alam G (2020). Pharmacological inhibition of CSF1R by GW2580 reduces microglial proliferation and is protective against neuroinflammation and dopaminergic neurodegeneration. FASEB journal: official publication of the Federation of American Societies for Experimental Biology.

[29] Chalmers SA, Wen J, Shum J, Doerner J, Herlitz L, Putterman C (2017). CSF-1R inhibition attenuates renal and neuropsychiatric disease in murine lupus. Clin Immunol.

[30] Martinez-Muriana A, Mancuso R, Francos-Quijorna I, Olmos-Alonso A, Osta R, Perry VH (2016). CSF1R blockade slows the progression of amyotrophic lateral sclerosis by reducing microgliosis and invasion of macrophages into peripheral nerves. Sci Rep.

[31] Courtine G, Bunge MB, Fawcett JW, Grossman RG, Kaas JH, Lemon R (2007). Can experiments in nonhuman primates expedite the translation of treatments for spinal cord injury in humans?. Nat Med.

[32] Friedli L, Rosenzweig ES, Barraud Q, Schubert M, Dominici N, Awai L (2015). Pronounced species divergence in corticospinal tract reorganization and functional recovery after lateralized spinal cord injury favors primates. Sci. Transl. Med.

[33] Noristani HN, They L, Perrin FE (2018). C57BL/6 and Swiss Webster mice display differences in mobility, gliosis, microcavity formation and lesion volume after severe spinal cord injury. Front Cell Neurosci.

[34] Le Corre M, Noristani HN, Mestre-Frances N, Saint-Martin GP, Coillot C, Goze-Bac C (2018). A novel translational model of spinal cord injury in nonhuman primate. Neurotherapeutics.

[35] Nair AB, Jacob S (2016). A simple practice guide for dose conversion between animals and human. J Basic Clin Pharm.

[36] Vinot N, Jouin M, Lhomme-Duchadeuil A, Guesnet P, Alessandri JM, Aujard F (2011). Omega-3 fatty acids from fish oil lower anxiety, improve cognitive functions and reduce spontaneous locomotor activity in a non-human primate. PLoS One.

[37] Noristani HN, Saint-Martin GP, Cardoso M, Sidiboulenouar R, Catteau M, Coillot C (2018). Longitudinal MRI analysis and histological characterization after spinal cord injury in two mouse strains with different functional recovery: gliosis as a key factor. J Neurotrauma.

[38] Coillot C, Sidiboulenouar R, Nativel E, Zanca M, Alibert E, Saint Martin G (2016). Signal modeling of a MRI ribbon solenoid coil dedicated to spinal cord injuries investigations. JSSS.

[39] Noristani HN, Lonjon N, Cardoso M, Le Corre M, Chan-Seng E, Captier G (2015). Correlation of *in vivo* and *ex vivo* (1)H-MRI with histology in two severities of mouse spinal cord injury. Front Neuroanat.

[40] Mytskaniuk V, Bardin F, Boukhaddaoui H, Rigneault H, Tricaud N (2016). Implementation of a Coherent Anti-Stokes Raman Scattering (CARS) system on a Ti:Sapphire and OPO laser based standard laser scanning microscope. J Vis Exp.

[41] Karnovsky MJ, Roots L (1964). A. Direct-coloring thiocholine method for cholinesterases. J Histochem Cytochem.

[42] Perrin FE, Lacroix S, Aviles-Trigueros M, David S (2005). Involvement of monocyte chemoattractant protein-1, macrophage inflammatory protein-1alpha and interleukin-1beta in Wallerian degeneration. Brain.

[43] Galli R, Sitoci-Ficici KH, Uckermann O, Later R, Mareckova M, Koch M (2018). Label-free multiphoton microscopy reveals relevant tissue changes induced by alginate hydrogel implantation in rat spinal cord injury. Sci Rep.

[44] Shi F, Zhu H, Yang S, Liu Y, Feng Y, Shi J (2009). Glial response and myelin clearance in areas of wallerian degeneration after spinal cord hemisection in the monkey Macaca fascicularis. J Neurotrauma.

[45] Brennan FH, Popovich PG (2018). Emerging targets for reprograming the immune response to promote repair and recovery of function after spinal cord injury. Curr Opin Neurol.

[46] David S, Greenhalgh AD, Kroner A (2015). Macrophage and microglial plasticity in the injured spinal cord. Neuroscience.

[47] Salter MW, Stevens B (2017). Microglia emerge as central players in brain disease. Nat Med.

[48] Masterson JC, O'Dea S (2007). 5-Bromo-2-deoxyuridine activates DNA damage signalling responses and induces a senescence-like phenotype in p16-null lung cancer cells. Anticancer Drugs.

[49] Ross HH, Levkoff LH, Marshall GP 2nd, Caldeira M, Steindler DA, Reynolds BA (2008). Bromodeoxyuridine induces senescence in neural stem and progenitor cells. Stem Cells.

[50] Michishita E, Nakabayashi K, Suzuki T, Kaul SC, Ogino H, Fujii M (1999). 5-Bromodeoxyuridine induces senescence-like phenomena in mammalian cells regardless of cell type or species. J Biochem.

[51] Schwartz ED, Hackney DB (2003). Diffusion-weighted MRI and the evaluation of spinal cord axonal integrity following injury and treatment. Exp Neurol.

[52] Vedantam A, Jirjis MB, Schmit BD, Wang MC, Ulmer JL, Kurpad SN (2014). Diffusion tensor imaging of the spinal cord: insights from animal and human studies. Neurosurgery.

[53] Saxena T, Deng B, Stelzner D, Hasenwinkel J, Chaiken J (2011). Raman spectroscopic investigation of spinal cord injury in a rat model. J Biomed Opt.

[54] Shi Y, Zhang D, Huff TB, Wang X, Shi R, Xu XM (2011). Longitudinal *in vivo* coherent anti-Stokes Raman scattering imaging of demyelination and remyelination in injured spinal cord. J Biomed Opt.

[55] Wang H, Fu Y, Zickmund P, Shi R, Cheng JX (2005). Coherent anti-stokes Raman scattering imaging of axonal myelin in live spinal tissues. Biophys J.

[56] Tamosaityte S, Galli R, Uckermann O, Sitoci-Ficici KH, Koch M, Later R (2016). Inflammation-related alterations of lipids after spinal cord injury revealed by Raman spectroscopy. J Biomed Opt.

[57] Zeng Y, Wang N, Guo T, Zheng Q, Wang S, Wu S (2018). Snx27 deletion promotes recovery from spinal cord injury by neuroprotection and reduces macrophage/microglia proliferation. Front Neurol.

[58] Shechter R, London A, Varol C, Raposo C, Cusimano M, Yovel G (2009). Infiltrating blood-derived macrophages are vital cells playing an anti-inflammatory role in recovery from spinal cord injury in mice. PLoS Med.

[59] Hamilton JA (2008). Colony-stimulating factors in inflammation and autoimmunity. Nat Rev Immunol.

[60] Hamilton JA, Whitty G, Masendycz P, Wilson NJ, Jackson J, De Nardo D (2008). The critical role of the colony-stimulating factor-1 receptor in the differentiation of myeloblastic leukemia cells. Mol Cancer Res.

[61] Pixley FJ, Stanley ER (2004). CSF-1 regulation of the wandering macrophage: complexity in action. Trends Cell Biol.

[62] Wang Y, Berezovska O, Fedoroff S (1999). Expression of colony stimulating factor-1 receptor (CSF-1R) by CNS neurons in mice. J Neurosci Res.

[63] Lalancette-Hebert M, Gowing G, Simard A, Weng YC, Kriz J (2007). Selective ablation of proliferating microglial cells exacerbates ischemic injury in the brain. J Neurosci.

[64] Grabert K, Michoel T, Karavolos MH, Clohisey S, Baillie JK, Stevens MP (2016). Microglial brain region-dependent diversity and selective regional sensitivities to aging. Nat Neurosci.

[65] Askew K, Li K, Olmos-Alonso A, Garcia-Moreno F, Liang Y, Richardson P (2017). Coupled proliferation and apoptosis maintain the rapid turnover of microglia in the adult brain. Cell reports.

[66] Shankaran M, Marino ME, Busch R, Keim C, King C, Lee J (2007). Measurement of brain microglial proliferation rates *in vivo* in response to neuroinflammatory stimuli: application to drug discovery. J Neurosci Res.

[67] Tay TL, Mai D, Dautzenberg J, Fernandez-Klett F, Lin G, Sagar (2017). A new fate mapping system reveals context-dependent random or clonal expansion of microglia. Nat Neurosci.

[68] Reu P, Khosravi A, Bernard S, Mold JE, Salehpour M, Alkass K (2017). The Lifespan and turnover of microglia in the human brain. Cell reports.

[69] Tonchev AB, Yamashima T, Zhao L, Okano H (2003). Differential proliferative response in the postischemic hippocampus, temporal cortex, and olfactory bulb of young adult macaque monkeys. Glia.

[70] Festa ED, Hankiewicz K, Kim S, Skurnick J, Wolansky LJ, Cook SD (2009). Serum levels of CXCL13 are elevated in active multiple sclerosis. Mult Scler.

[71] Kuenz B, Lutterotti A, Ehling R, Gneiss C, Haemmerle M, Rainer C (2008). Cerebrospinal fluid B cells correlate with early brain inflammation in multiple sclerosis. PLoS One.

[72] Okuneva O, Li Z, Korber I, Tegelberg S, Joensuu T, Tian L (2016). Brain inflammation is accompanied by peripheral inflammation in Cstb (-/-) mice, a model for progressive myoclonus epilepsy. J Neuroinflammation.

[73] Chen F, Li X, Li Z, Qiang Z, Ma H (2020). Altered expression of MiR-186-5p and its target genes after spinal cord ischemia-reperfusion injury in rats. Neurosci Lett.

[74] Chen F, Li X, Li Z, Zhou Y, Qiang Z, Ma H (2020). The roles of chemokine (C-X-C motif) ligand 13 in spinal cord ischemia-reperfusion injury in rats. Brain Res.

[75] Chen F, Wang D, Jiang Y, Ma H, Li X, Wang H (2021). Dexmedetomidine postconditioning alleviates spinal cord ischemia-reperfusion injury in rats via inhibiting neutrophil infiltration, microglia activation, reactive gliosis and CXCL13/CXCR5 axis activation. Int J Neurosci.

[76] Jones LL, Yamaguchi Y, Stallcup WB, Tuszynski MH (2002). NG2 is a major chondroitin sulfate proteoglycan produced after spinal cord injury and is expressed by macrophages and oligodendrocyte progenitors. J Neurosci.

[77] Huang W, Bai X, Meyer E, Scheller A (2020). Acute brain injuries trigger microglia as an additional source of the proteoglycan NG2. Acta Neuropathol Commun.

