# Supplementary material for: Inhibiting microglia proliferation after spinal cord injury improves recovery in mice and nonhuman primates
Source: Theranostics. 2021 Jul 31;11(18):8640–59. doi: 10.7150/thno.61833 (PMC8419033; doi:10.7150/thno.61833)
Supplement: Supplementary file 1 — Supplementary figures and tables. [file thnov11p8640s1.pdf]

Supplementary data

Inhibiting microglia proliferation after spinal cord injury improves recovery in mice and nonhuman primates

Gaëtan Poulen, Emilie Aloy, Claire M. Bringuier, Nadine Mestre-Francés, Emaëlle V.F. Artus, Maïda Cardoso, Jean-Christophe Perez, Christophe Goze-Bac, Hassan Boukhaddaoui, Nicolas Lonjon, Yannick N. Gerber and Florence E. Perrin

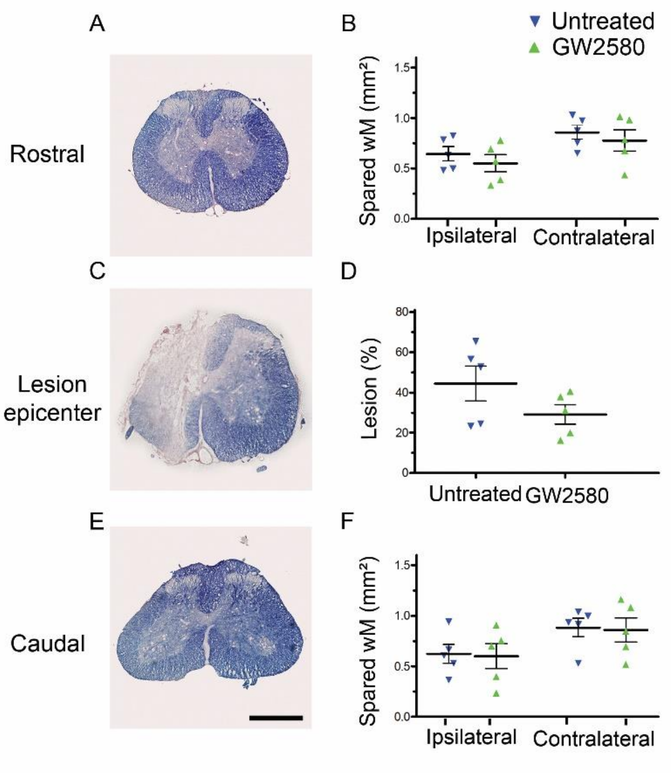

<u>**Supplementary Figure 1:**</u> **Transient CSF1R blockade after lateral spinal cord hemisection in nonhuman primates modifies lesion extension**

Bright-field micrographs displaying Luxol fast blue and neutral red stained axial sections rostral (**A**) within (**C**), and caudal (**E**) to the lesion site at 3 months after SCI in a GW2580-treated nonhuman primate. Luxol-based quantifications of the spared white matter (WM) rostral (**B**) and caudal (**F**) to the lesion site as well as the percentage of damaged tissue at the epicenter (**D**). Scale bar: 600µm. In all graphs, results for untreated nonhuman primates are in blue and GW2580-treated in green. Each point for a given animal represents the mean of 16 slices (**B&F**) Data are mean ± SEM. Number of injured *Microcebus murinus*: untreated n=5 and GW2580-treated for 2 weeks n=5.

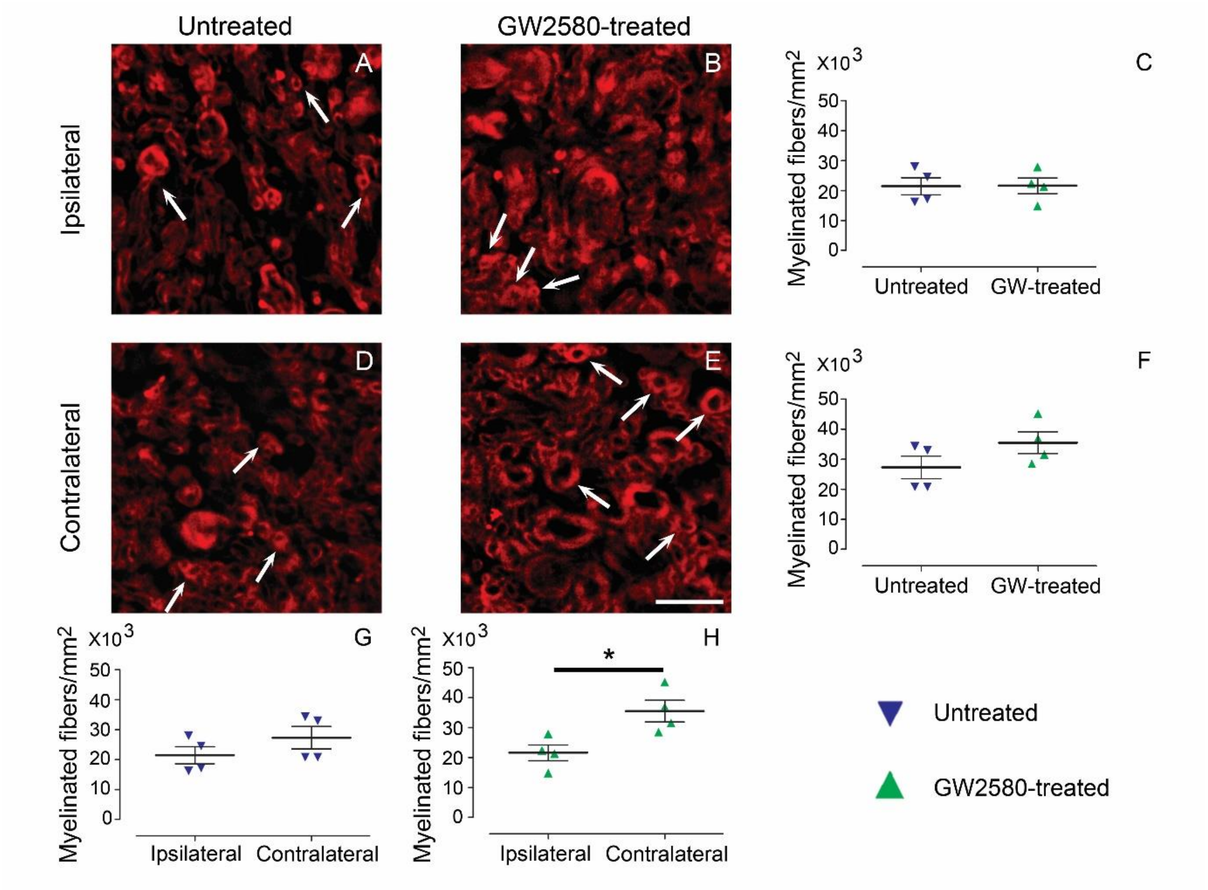

<u>Supplementary Figure 2 :</u> **Transient CSF1R blockade after lateral spinal cord hemisection in nonhuman primates modifies outcomes on myelinated fibers**

Photographs of fluoromyelin staining of untreated (**A&D**) and GW2580-treated (**B&E**) *Microcebus murinus*. Pictures of axial section of the thoracic spinal cord taken in the lateral *funiculus* 1.68 mm caudal to the lesion epicenter on the ipsilateral (**A&B**) and contralateral (**D&E**) sides of the lesion. Quantification of intact myelin sheaths on the ipsilateral (**C**) and contralateral (**F**) sides of the lesion in untreated and GW2580-treated animals 3 months after SCI. Intact myelin sheaths quantification on the ipsilateral and contralateral sides of the lesion in untreated (**G**) and GW2580-treated (**H**) animals. In all graphs, results for untreated nonhuman primates are in blue and GW2580-treated in green. Four animals selected randomly in each group (untreated and GW2580-treated) and 2 pictures (ipsilateral and contralateral) were quantified per lemur. Data are mean ± SEM per group. Student's unpaired t-test, $*p < 0.05$. Scale bars: **A**-**D**, 10µm. Arrows (**A&D** and **B&E**) point to intact myelin sheaths.

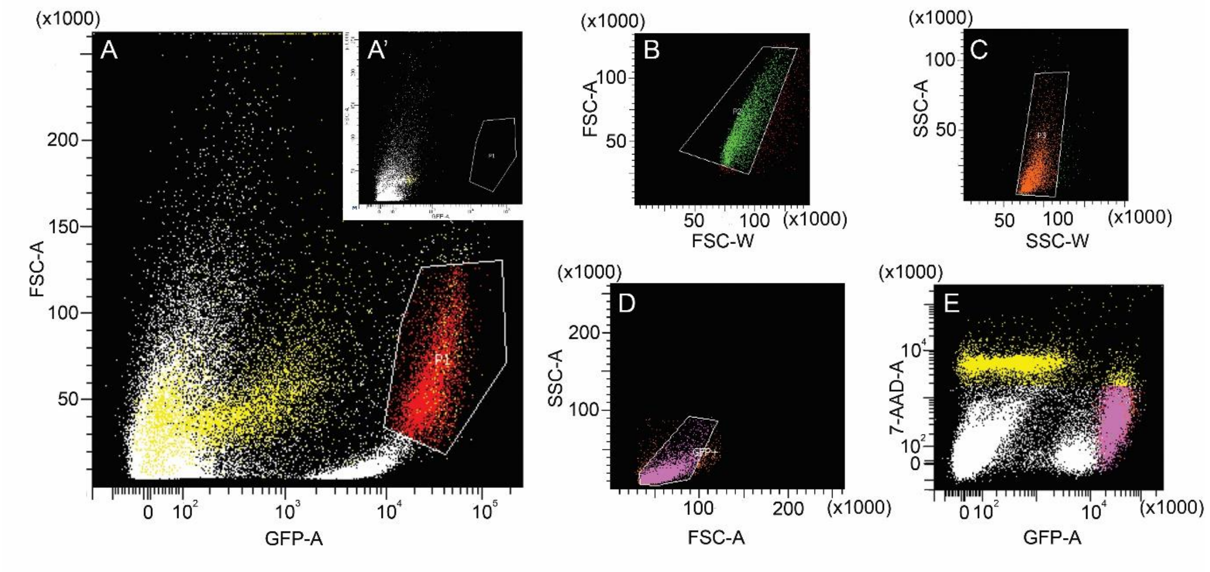

<u>Supplementary Figure 3:</u> gating strategy for FACS

Representative flow cytometry from injured CX3CR1$^{+/GFP}$ male mice displaying eGFP^{high} expressing microglia designed as P1 (**A**) as compared to FACS profile of injured “none GFP” mice (**A’**). Gating strategy based on doublet exclusion (**B**) and morphology (**C**) for selected eGFP^{high} expressing cell population. Flow cytometry profile displaying sorted cells, designed as “GFP+”, that correspond to the eGFP^{high}-expressing microglia further analyzed using transcriptomic (**D**) excluding dead cells stained with 7AAD (**E**, appear in yellow). FSC: Forward Scatter; SSC: Size Scatter; A: Area; W: Width.

<u>Supplementary Table 1.</u> Database of differential expression comparison of microglia from GW2580-treated and untreated mice at 1 week after spinal cord hemisection. p-value with FDR≤0.05 and FC≥2.

Gene	Description	GO Process	Fold Change	p-value with FDR
Cd38	CD38 antigen	Negative regulation of neuron projection	-7.71	0.0024
Cd40	CD40 antigen	Immune process	-3.48	0.0124
Cxcl13	Chemokine (C-X-C motif) ligand 13	Immune process	-3.34	0.0214
Pf4	Platelet factor 4	Inflammatory response	-3.3	0.0155
Emilin2	Elastin microfibril interfacer 2		-3.12	0.0057
Lyz1	Lysozyme 1	Defense response to bacterium	-2.89	0.0402
Msr1	Macrophage scavenger receptor 1	Positive regulation of macrophage derived foam cell differentiation	-2.82	0.0024
Fn1	Fibronectin 1	Positive regulation of cell proliferation regulation	-2.80	0.0087
Cspg4	Chondroitin sulfate proteoglycan 4	Cell population proliferation, glial cell migration	-2.74	0.0107
Adm	Adrenomedullin	Positive regulation of cell proliferation regulation	-2.59	0.0327
Cybb	Cytochrome b-245. beta polypeptide	Inflammatory response	-2.27	0.0024
Pltp	Phospholipid transfer protein	Lipid metabolic process	-2.27	0.0201
Gpx3	Glutathione peroxidase 3	Response to oxidative stress	-2.26	0.0057
Lyz2	Lysozyme 2	Killing of cells of other organism	-2.12	0.0134
Itsn1	Intersectin 1 (SH3 domain protein 1A)	Brain development	-2.07	0.0407
Gpnmb	Glycoprotein (transmembrane) nmb	Positive regulation of cell migration	-2.07	0.0051
Kcnk12	Potassium channel. subfamily K. member 12	Potassium ion transmembrane transport	2.06	0.0377
Srpk3	Serine/arginine-rich protein specific kinase 3	Cell differentiation	2.08	0.0104
Sdk1	Sidekick cell adhesion molecule 1	Cell adhesion	2.32	0.0191

<u>Supplementary Table 2.</u> Enrichment analysis in the comparison of microglia from GW2580-treated and untreated mice at 1 week after spinal cord hemisection. p-value with FDR≤0.05 and FC≥2.

Rank	Molecular function	p-value with FDR	Genes in data vs total genes in the pathway	Genes
**1**	CXCR3 chemokine receptor binding	7.314E-4	2/5	*Cxcl13, Pf4*
**2**	Heparin binding	1.150E-3	4/221	*Gpnmb* (*Osteoactivin*), *fibronectin, Cxcl13, Pf4*
**3**	Lysozyme activity	6.219E-19	2/13	*Lyz1, Lyz2*

Rank	Processes	p-value/ FDR	Genes in data vs total genes in the pathway	Genes
**1**	Inflammatory response	5.588E-05	8/874	*Adrenomedullin, Cd40, Cspg4, fibronectin, Cxcl13, Pf4, Cybb, Lyzozym*
**2**	Regulation of angiogenesis	1.487E-04	6/484	*Adrenomedullin, Cd40, Cxcl13, Pf4, Cybb, Gpnmb* (*Osteoactivin*)
**3**	Regulation of developmental process	1.487E-04	12/3978	*Adrenomedullin, Cd38, Cd40, Cspg4, fibronectin, Cxcl13, Cybb, Gpnmb* (*Osteoactivin*), *Gpx, Intersectin, Msr1, Intersectin*
**4**	Response to other organism	1.487E-04	10/2427	*Adrenomedullin, Cd40, fibronectin, Cxcl13, Cybb, Gpx, Gpx3, Lysozyme, Lyz2, Pf4*
**5**	Response to external biotic stimulus	1.487E-04	10/2431	*Adrenomedullin, Cd40, fibronectin, Cxcl13, Cybb, Gpx, Gpx3, Lysozyme, Lyz2, Pf4*
